# Intrinsic linker flexibility in an anionic metal–organic framework electrolyte for selective lithium-ion conduction

**DOI:** 10.1039/d6sc06134f

**Published:** 2026-08-28

**Authors:** Zina Deriche, Vallabha Rao Rikka, Isaac Metcalf, Ahad Hussain Javed, Satya Prakash Suman, Moneer Alenezi, Aditya Mohite, Sibani Lisa Biswal, Stavroula Kampouri

**Affiliations:** a Department of Chemical and Biomolecular Engineering, Rice University Houston TX 77005 USA; b Rice Advanced Materials Institute (RAMI), Rice University Houston TX 77005 USA; c Chemistry Department, Rice University Houston TX 77005 USA; d Electrochemical Safety Research Institute (ESRI) Houston TX 77023 USA

## Abstract

Although solid-state batteries promise improved safety and higher energy density, their performance is fundamentally constrained by solid electrolytes that fail to reconcile fast, selective Li^+^ transport with conformal interfacial contact. Here, we report ZnBTCA, an anionic zinc-based metal–organic framework constructed from the flexible aliphatic linker 1,2,3,4-butanetetracarboxylic acid, introducing intrinsic framework compliance. Ion exchange converts Na^+^-ZnBTCA to its Li^+^ form, enabling highly Li^+^-selective ionic conduction within a mechanically soft framework (Young's modulus &ap; 4.6 GPa) that promotes conformal Li|electrolyte contact and reduces interfacial impedance. ZnBTCA exhibits Li^+^ conductivities of 3.53 × 10^−5^ S cm^−1^ at 20 °C and 1.87 × 10^−4^ S cm^−1^ at 60 °C (*E*_a_ = 0.36 eV), together with a high Li^+^ transference number (*t*_Li^+^_ = 0.79). This combination of structural compliance and selective Li^+^ conduction enables stable interfacial behavior, demonstrated by ∼300 h of Li|ZnBTCA@5-PEO|Li cycling at 0.2–1.0 mA cm^−2^ with stable voltage polarization. To our knowledge, this represents the first aliphatic-based MOF electrolyte and establishes linker flexibility coupled with framework anionicity as a general design strategy for mechanically adaptive, high Li^+^ transport MOF electrolytes in next-generation solid-state batteries.

## Abstract 2

ZnBTCA, a soft anionic MOF, facilitates targeted for Li^+^ transport serves as a promising solid-state battery electrolyte.

## Graphical abstract

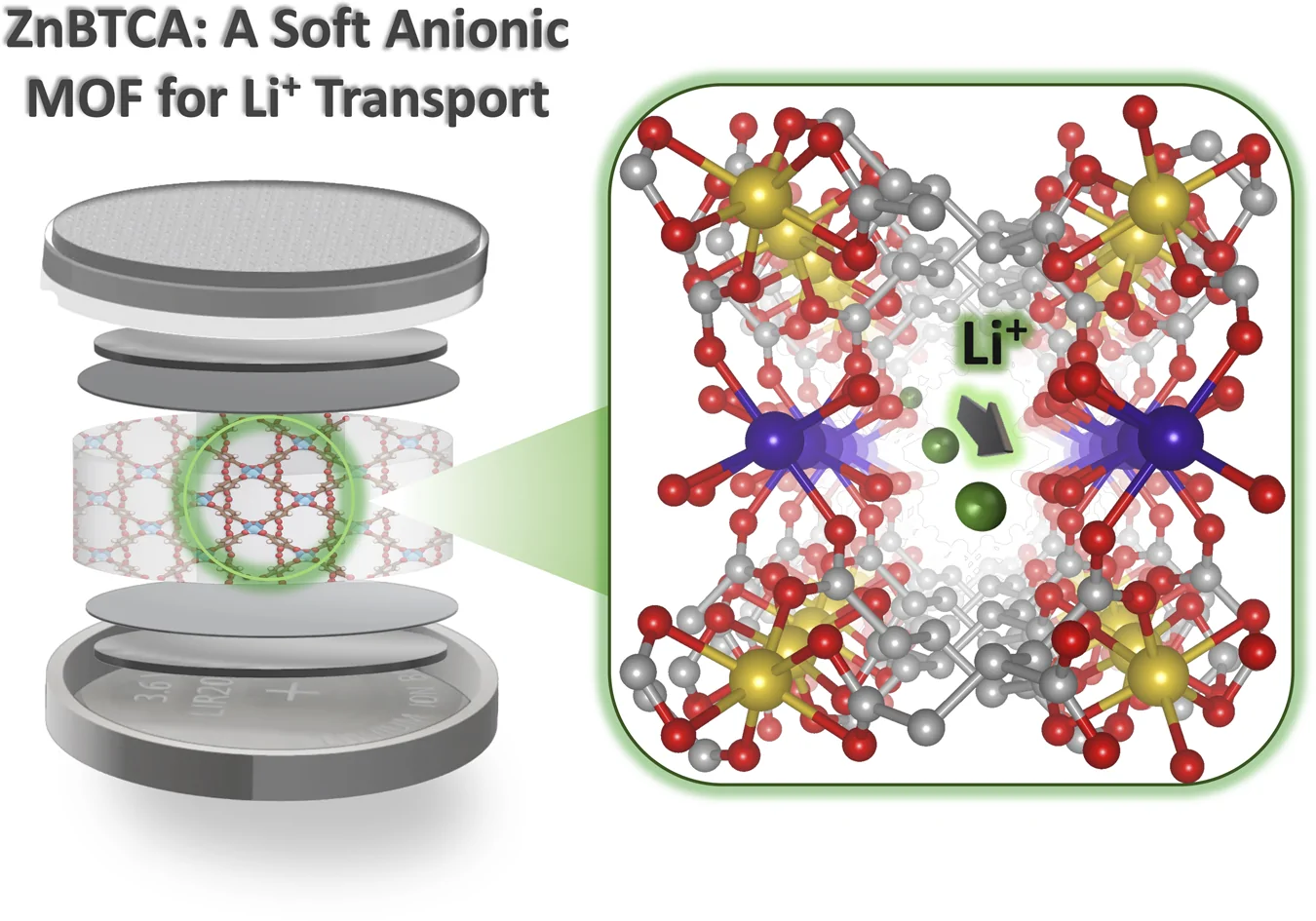

## 1 Introduction

The urgent need for safer energy storage systems with higher energy density drives the rapid development of solid-state batteries (SSBs).^1,2^ Traditional lithium-ion (Li^+^) batteries rely on flammable liquid electrolytes that pose safety risks such as leakage, volatility, and Li dendrite formation.^2,3^ In contrast, solid-state electrolytes (SSEs) offer improved thermal stability, enhanced mechanical robustness, and superior compatibility with Li metal anodes, making them promising candidates for next-generation batteries.^4,5^ Nonetheless, achieving high Li^+^ conductivity in the solid state, particularly at room temperature, remains a persistent challenge that limits their practical application.^6^ Beyond bulk conductivity, designing solid electrolytes that simultaneously maintain mechanical compliance and selective ion transport remains a central and unresolved challenge.^7^

Metal–organic frameworks (MOFs) have emerged as attractive candidates for solid electrolytes due to their permanent porosity, tunable chemistry, and diverse crystalline architectures.^8–11^ The well-defined pore structures of MOFs can serve as ion-conduction channels, enabling them to mimic certain transport properties of liquids while maintaining the mechanical integrity of solids. Moreover, their modular design enables systematic control over pore geometry and chemical functionality, providing a uniquely powerful platform for elucidating structure–transport relationships in solid-state ion conduction.^7^

Indeed, several MOF-based solid electrolytes have achieved room-temperature conductivities on the order of 10^−4^–10^−5^ S cm^−1^ together with moderate to high Li^+^ transference numbers (Table S3).^10,13–18^ However, despite these promising transport metrics, conventional MOF-based electrolytes often suffer from interfacial contact issues and constrained local framework dynamics, both of which hinder efficient ionic transport.^7^ These limitations largely stem from the intrinsic structural rigidity of the frameworks, as most MOFs rely on rigid aromatic linkers, such as terephthalic acid and its derivatives, that demonstrate limited conformational flexibility. Recent strategies have explored linker functionalization and the formation of composites with polymers.^18^ However, these approaches frequently compromise the intrinsic porosity of MOFs, a defining feature for effective ion transport. Thus, introducing framework flexibility without compromising porosity or ion selectivity remains an unresolved design challenge.

Here, we propose an alternative design principle: introducing intrinsic framework flexibility through the incorporation of an aliphatic linker as a MOF building block to enhance both bulk ion transport and interfacial adaptability. Aliphatic linkers containing sp^3^ C–C σ bonds provide rotational freedom absent in π-conjugated aromatic systems, reducing intrinsic framework stiffness and enabling localized segmental motion.^19^ This mechanically encoded flexibility creates dynamically adaptive ion-conduction channels while preserving crystalline order. Such dynamic behavior may facilitate cooperative Li^+^ migration and improve conformal electrode–electrolyte contact, analogous to that observed in conventional non-porous polymers.^20^ We therefore hypothesize that coupling linker-induced mechanical flexibility with an anionic framework architecture can simultaneously enhance Li^+^ transport and interfacial behavior, offering a new design paradigm for MOF-based solid electrolytes.

In this work, we report a zinc-based anionic MOF constructed from the aliphatic linker 1,2,3,4-butanetetracarboxylic acid (BTCA), herein referred to as ZnBTCA.^11^ Spectroscopic analyses reveal that the anionic ZnBTCA is charge-balanced by sodium cations (Na^+^), which can be readily exchanged with mobile Li^+^. The resulting material combines structural adaptability with mechanical softness and is composed of earth-abundant, low-cost components. To the best of our knowledge, this is the first demonstration of an aliphatic MOF functioning as a solid-state electrolyte. By correlating structure, mechanical properties, ionic conductivity, and long-term cycling performance, we demonstrate that linker flexibility, coupled with framework anionicity, defines a new axis for rational electrolyte design beyond conventional pore- or compositional-tuning. We hope this principle establishes a generalizable strategy for designing mechanically adaptive, ion-conductive MOFs for solid-state batteries.

## 2 Results and discussion

### 2.1 Crystal structure and framework description

ZnBTCA was synthesized in aqueous media, yielding high-quality colorless crystals approximately 0.7 mm in size (detailed synthetic information is provided in Section S1.2 of the SI). Single-crystal X-ray diffraction (SC-XRD) and powder X-ray diffraction (PXRD) analysis, as presented in Fig. S1, were used to determine the structure of the ZnBTCA framework, which was previously reported.^10^ The structure consists of two crystallographically distinct Zn sites (Zn_1_ and Zn_2_) bridged by BTCA linkers. Zn_1_ is pseudo-octahedrally coordinated with six oxygen atoms, two of which stem from two BTCA linkers connected in a monodentate (*κ*_1_) fashion, and the other two are assigned to water or hydroxyl groups (Fig. 1(I)). Zn_2_ exhibits a distorted octahedral geometry, coordinated by both chelating (*κ*_2_) and monodentate carboxylate groups (Fig. 1(II)). The BTCA ligands act as µ_6_-bridging units, connecting the Zn^2+^ centers into a three-dimensional framework featuring one-dimensional channels along the *c*-axis (Fig. 1).

**Fig. 1:**
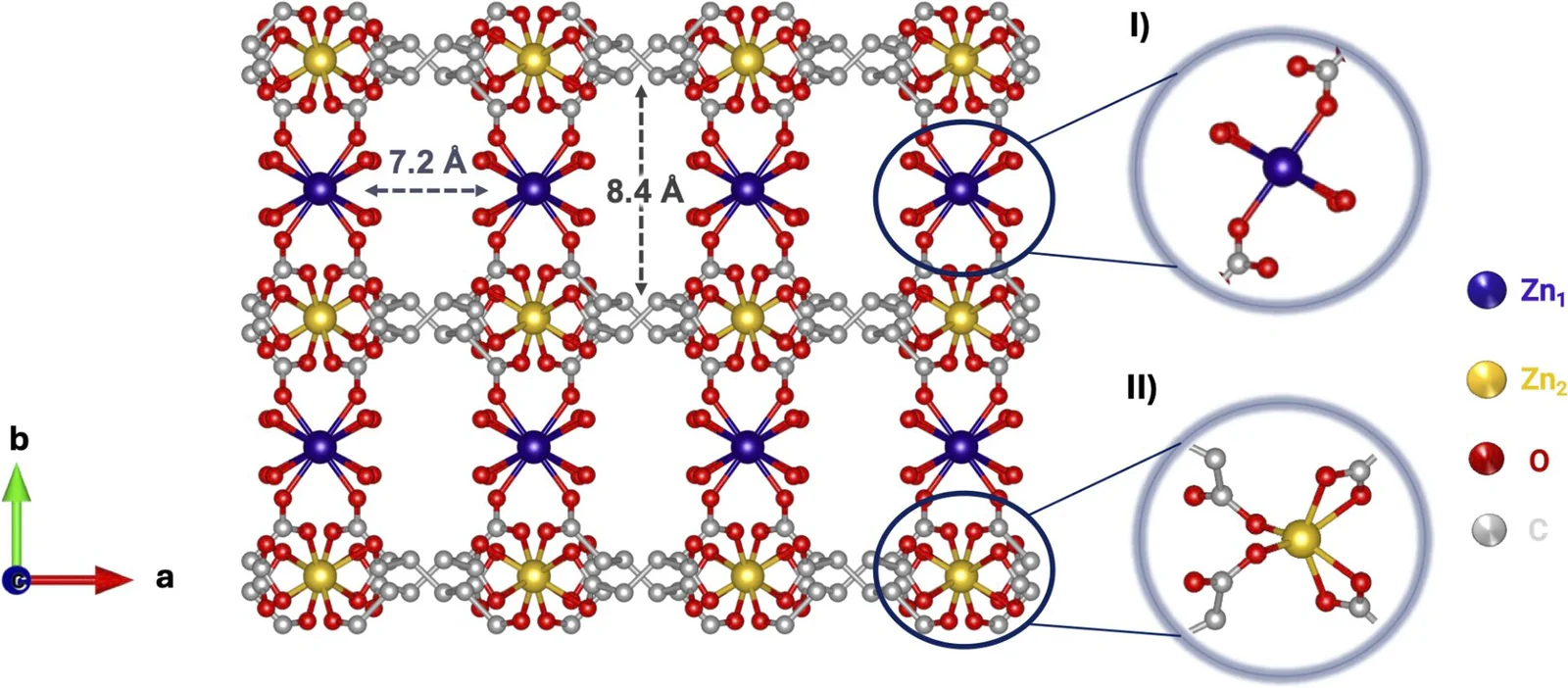
Crystal structure of ZnBTCA viewed along the *c* axis, showing three-dimensional connectivity and pore arrangement in the a and b plane. The two crystallographically distinct Zn sites are highlighted: (I) Zn_1_ (purple), has two BTCA linkers connected in a monodentate (*κ*_1_) fashion, and the remaining are assigned to water or hydroxyl groups; (II) Zn_2_ (yellow), which exhibits a distorted octahedral geometry, coordinated by two chelating (*κ*_2_) and two monodentate carboxylate groups. Carboxylate oxygens are red, and linker carbons are grey. The rectangular channel array exhibits pore-to-pore separations of 7.2 Å (along the *a* axis) and 8.4 Å (along the *b* axis) as indicated. Hydrogen atoms are omitted for clarity.

### 2.2 Revised chemical formula: evidence for an anionic framework

While the previously reported structure was described as a neutral composition [Zn_2_(BTCA)(H_2_O)_4_]·(H_2_O), our experimental data indicate a different charge balance. Despite the crystallographically observed one-dimensional channels, ZnBTCA exhibits a Type III-like N_2_ isotherm with limited uptake and a sharp increase near *P*/*P*_0_ = 1, consistent with weak N_2_-framework interactions and condensation within interparticle voids (Fig. S2). A geometric surface-area calculation performed on the guest-free SC-XRD structure gives a theoretical accessible surface area of 315 m^2^ g^−1^, indicating that the limited N_2_ uptake reflects restricted accessibility rather than an absence of structural porosity. This behavior could be indicative of charge-balancing species partially occupying the pore channels. Fourier transform infrared spectroscopy (FTIR) spectra collected before and after thermal activation at 120 °C under vacuum show a decrease in the broad O–H stretching band at approximately 3100 cm^−1^ and the H–O–H bending vibration at approximately 1650 cm^−1^, which is interpreted qualitatively as evidence of reduced water content (Fig. S3). However, a residual shoulder in the O–H region persists after activation, indicating the presence of coordinated water or hydroxyl groups that remain bound to the framework.

Further evidence for an intrinsically anionic framework is provided by elemental and surface analyses. X-ray photoelectron spectroscopy (XPS) (Fig. S4) and energy dispersive spectroscopy (EDS) (Fig. S5) consistently revealed the presence of Na^+^ in ZnBTCA, even after extensive washing with boiling water and methanol. The persistence of Na^+^ indicates that these cations act as extra-framework charge balancers residing within the 1D channels. Inductively coupled plasma optical emission spectroscopy (ICP-OES) (Fig. S6 and Table S1), revealed a Na : Zn molar ratio of 1.27, suggesting that the Zn_1_ coordination sites are occupied by both neutral H_2_O molecules and hydroxyl groups that compensate for the framework charge. On the basis of these findings, we revise the empirical formula of ZnBTCA to an anionic composition, [Zn_2_(BTCA)(H_2_O)_1.5_ (OH)_2.5_]·Na_2.5_].

Post-synthetic ion exchange experiments were performed by soaking ZnBTCA in LiTFSI (Lithium bis(trifluoro-methanesulfonyl)imide) solutions prepared in anhydrous acetonitrile. Successful incorporation of Li^+^ was supported by FTIR spectroscopy (Fig. S8), where the asymmetric and symmetric stretching vibrations of the carboxylate groups (∼1550 and ∼1400 cm^−1^, respectively) exhibited noticeable red-shifts consistent with changes in the local carboxylate environment following Li^+^ exchange. ICP-OES analysis of samples soaked for different durations further confirmed time-dependent Li^+^ incorporation and a decrease in the residual Na^+^ content of the framework. The 24 h exchanged sample contained approximately Na_0.15_ Li_2.55_ per Zn_2_ formula unit, corresponding to ∼95% Li^+^ among the mobile charge-balancing cations (Tables S4 and S5). Concentration-dependent ICP-OES measurements further showed increasing Li^+^ incorporation with increasing LiTFSI concentration, approaching a high degree of exchange at concentrations of 1 M and above (Table S6). Notably, the PXRD pattern of Li-exchanged ZnBTCA remained unchanged relative to the pristine material, confirming that the anionic framework remains structurally intact upon cation exchange.

### 2.3 Framework softness and mechanical adaptability

The incorporation of the aliphatic BTCA linker into a MOF produces a mechanically soft and structurally dynamic solid. Nanoindentation experiments, featured in Fig. 2, revealed that ZnBTCA possesses a Young's modulus of 4.6 ± 0.2 GPa, calculated by using the Oliver-Pharr method (eqn (S10)).^18,22^ This value places ZnBTCA among the softer reported MOFs (Table S2).^23–26^ This reduced stiffness may be advantageous in electrochemical applications, as a softer lattice can conform more intimately to electrode surfaces, improving interfacial contact and reducing interfacial impedance during cycling. Beyond interfacial effects, the relatively soft framework may help accommodate local changes associated with Li^+^ coordination and transport while maintaining the overall crystalline structure, as supported by the unchanged PXRD pattern after Li^+^ exchange (Fig. S1). For comparison, Al-fumarate, another non-aromatic carboxylate MOF, exhibited a Young's modulus of 5.7 GPa, indicating a similarly soft mechanical response (Fig. S13).

**Fig. 2:**
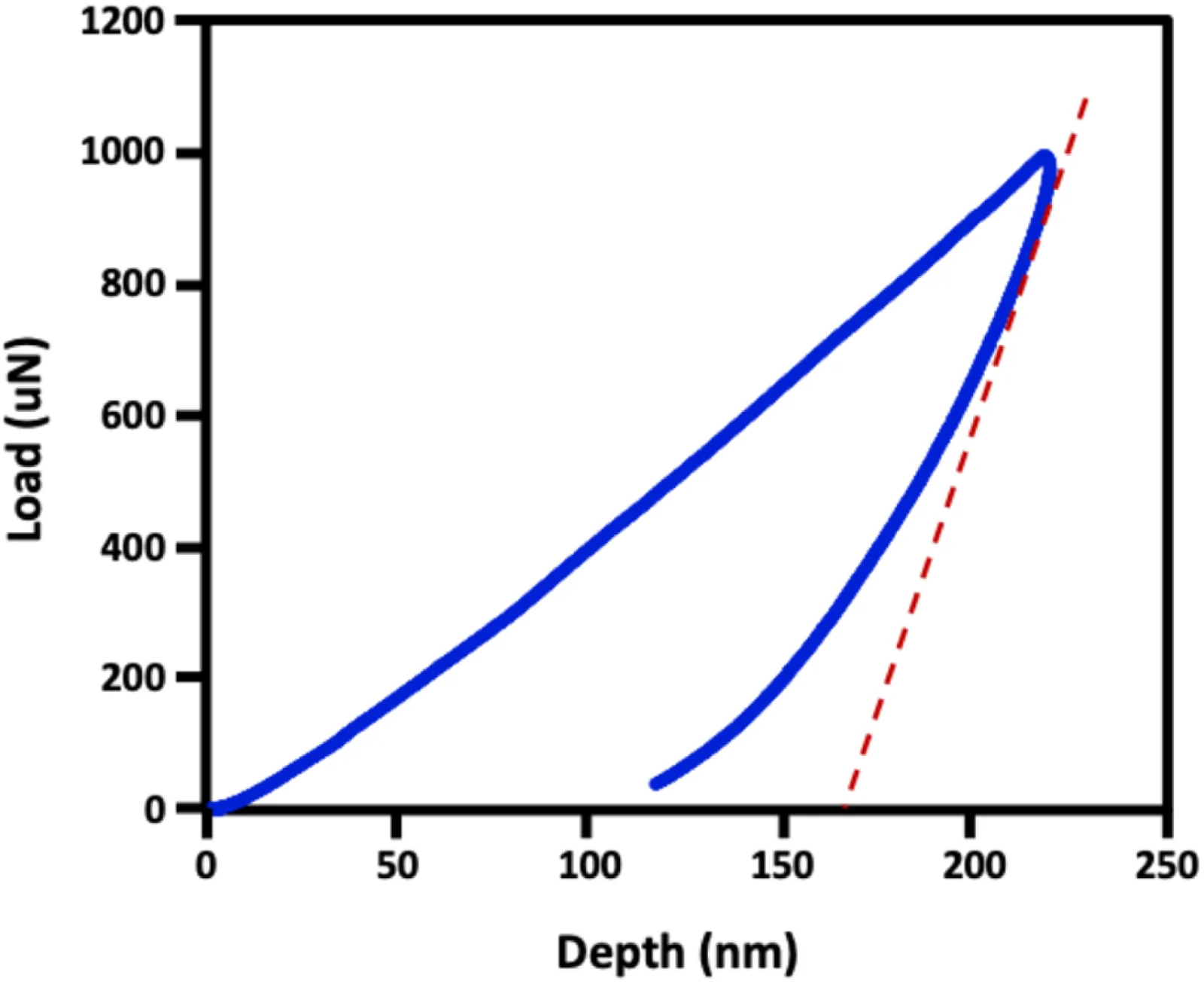
Nanoindentation load-displacement curve for ZnBTCA single crystals. The loading (blue solid line) and unloading (red dashed line) segments reveal the material's elastic recovery behaviour. The shallow unloading slope and maximum indentation depth reflect the framework's mechanical softness, corresponding to a reduced modulus of approximately 5.06 GPa, a Young's modulus of 4.6 GPa, and a hardness of 0.6 GPa, indicative of the flexible aliphatic linker environment.

### 2.4 Ionic conductivity and Arrhenius analysis

Ionic conductivity (*σ*) was determined using stainless-steel (SS) blocking electrodes in a Swagelok cell over 20–100 °C.^12,21^ The room-temperature conductivity was 3.53 × 10^−5^ S cm^−1^ and increased progressively to 6.44 × 10^−5^, 1.87 × 10^−4^, 3.79 × 10^−4^, and 6.46 × 10^−4^ S cm^−1^ at 40, 60, 80, and 100 °C, respectively (Fig. 3(I–III) and S9). To evaluate the effect of crystal size, ZnBTCA crystals were reduced from approximately 0.7 mm to approximately 0.01 mm, and the size-reduced sample exhibited a room-temperature effective ionic conductivity of 2.33 × 10^−5^ S cm^−1^, which is on the same order of magnitude as that of the as-synthesized crystals (Fig. S10). These pellet-based measurements are therefore reported as effective ionic conductivities and may include contributions from inter-particle and grain-boundary transport. The Arrhenius fit of ln(*σ*) *vs.* 1000/*T* was linear across this range, giving an activation energy of 0.36 eV (Fig. 3(IV)). The low activation energy (<0.4 eV) indicates thermally activated ionic transport in the ZnBTCA pellets.^7,10^

**Fig. 3:**
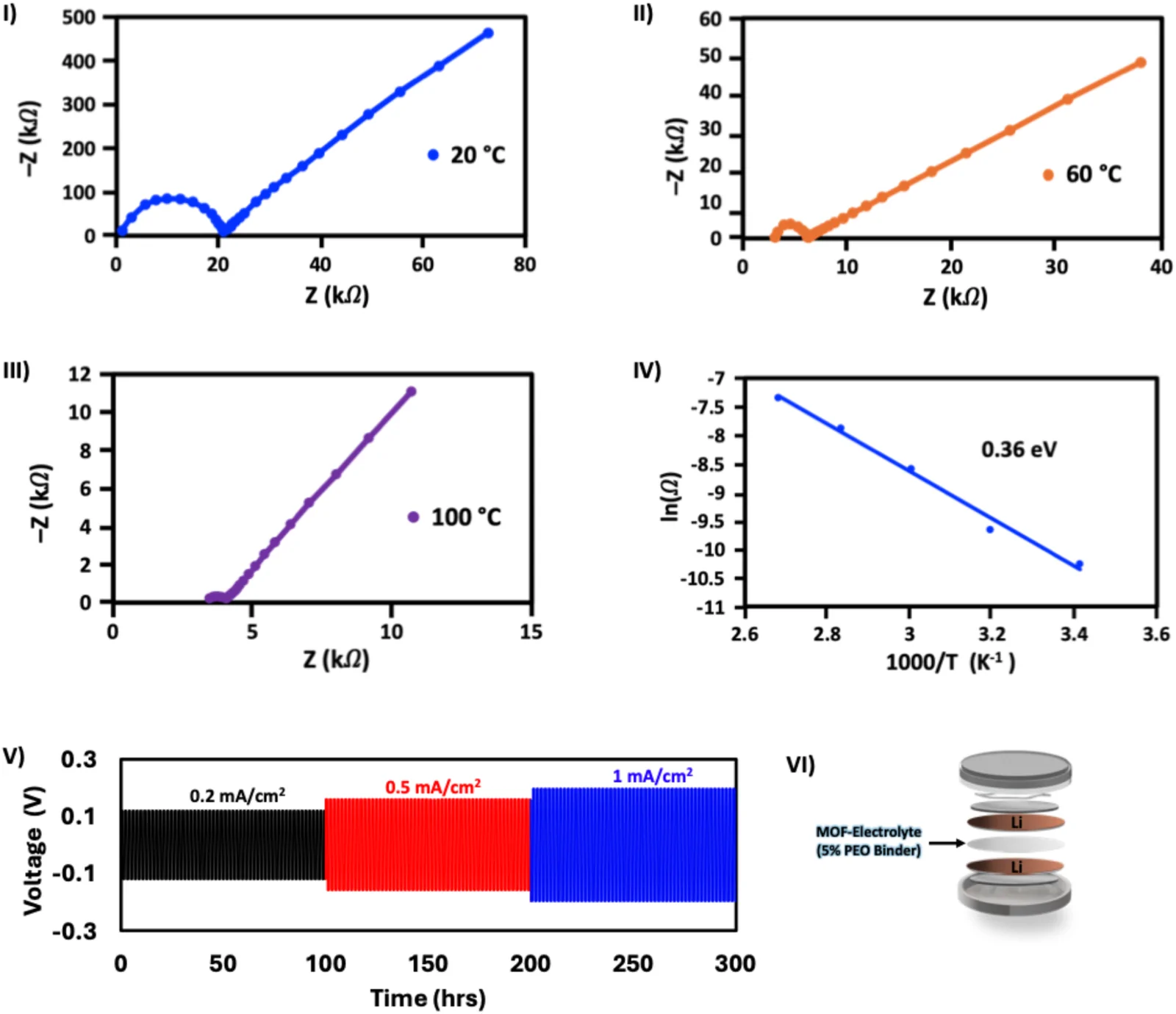
(I–III) Nyquist plots of ZnBTCA measured at 20 °C, 60 °C, and 100 °C, respectively, using stainless steel (SS) blocking electrodes. All spectra exhibit a well-defined high-frequency semicircle corresponding to the effective pellet/electrolyte resistance, followed by a low-frequency Warburg tail indicative of ion diffusion processes. (IV) Arrhenius plot of ionic conductivity derived from temperature-dependent impedance measurements, with a linear fit yielding an activation energy of 0.36 eV. (V) Galvanostatic cycling of symmetric cells at stepwise increasing current densities of 0.2, 0.5, and 1.0 mA cm^−2^. The voltage profiles show stable polarization over nearly 300 h of continuous operation, demonstrating reversible and uniform Li plating/stripping behavior. (VI) Schematic illustration of the symmetric cell configuration used for electrochemical evaluation, where the MOF electrolyte (5% PEO binder) is sandwiched between two lithium metal electrodes.

To probe interfacial behavior with Li metal, we fabricated Li|ZnBTCA|Li symmetric coin cells. Using potentiostatic polarization and the Evans–Vincent–Bruce method (eqn (S3) and Fig. S11), the Li^+^ transference number was determined to be 0.79, which is notably higher than typical values for Li^+^ in liquid electrolytes (≈0.4), and among the highest in the field of MOFs (Table S3).^10,13–18^ This indicates that ionic transport in ZnBTCA is predominantly carried by Li^+^. As shown in Fig. S11, the resistance decreases after polarization, suggesting interfacial conditioning during the initial cycling. This behavior may arise from improved Li–electrolyte contact and redistribution of mobile Li^+^ ions at the interface, facilitated by the mechanical compliance of the MOF. These measurements support predominantly Li^+^-mediated macroscopic transport but do not resolve local Li^+^ environments or microscopic hopping pathways, which could be further investigated using solid-state ^7^Li NMR.

### 2.5 Electrochemical cycling performance

A freestanding solid electrolyte membrane was fabricated using ZnBTCA with 5% polyethylene oxide (PEO) as a binder. Galvanostatic cycling was performed on Li|ZnBTCA@5-PEO|Li symmetric cells. At a current density of 0.2 mA cm^−2^, the cell exhibited steady operation for ∼100 h, after which the current density was sequentially increased to 0.5 mA cm^−2^ and 1.0 mA cm^−2^, with continued stable cycling for a total duration of nearly 300 h (Fig. 3(V and VI)).

The cell maintained stable cycling without evidence of short-circuiting under the tested conditions.^11,14^ This stability may arise from the mechanical softness of ZnBTCA, which can accommodate local strain and promote favorable electrode–electrolyte contact, together with interfacial and grain–boundary pathways that support stable Li plating/stripping under the tested conditions.

## 3 Conclusions

We report the rational design and electrochemical validation of ZnBTCA, a zinc-based anionic MOF constructed from a flexible aliphatic linker (BTCA) that imparts intrinsic mechanical softness to the framework. Upon Li^+^ exchange, ZnBTCA enables predominantly Li^+^-selective conduction within a mechanically softer lattice relative to conventional aromatic MOFs (Young's modulus = 4.6 GPa), exhibiting a room-temperature ionic conductivity of 3.53 × 10^−5^ S cm^−1^, increasing to 6.46 × 10^−4^ S cm^−1^ at 100 °C, with a low activation energy (*E*_a_ = 0.36 eV). The high Li^+^ transference number (*t*_Li^+^_ = 0.79) confirms predominantly Li^+^-selective transport, which may help reduce concentration polarization and contribute to stable electrochemical behavior. Symmetric Li|ZnBTCA@5-PEO|Li cells demonstrate stable cycling for 300 h with stable voltage polarization and reduced interfacial resistance, highlighting the benefits of mechanically adaptive frameworks.

Collectively, these findings demonstrate that coupling linker softness with framework anionicity provides a powerful strategy to simultaneously promote selective Li^+^ transport and electrode–electrolyte interfacial stability in MOF-based electrolytes. To our knowledge, ZnBTCA represents the first aliphatic-linker-based MOF electrolyte, establishing linker flexibility as a powerful strategy for developing mechanically adaptive, high-Li^+^-selective solid-state electrolytes for next-generation batteries.

## Author contributions

Z. D.: conceptualization (lead); methodology; investigation; validation; formal analysis; data curation; visualization; writing – original draft. V. R. R.: investigation. I. M.: investigation. A. H. J.: investigation. S. P. S.: investigation. M. A.: investigation. A. M.: writing – review & editing. S. L. B.: conceptualization; supervision; writing – review & editing. S. K.: conceptualization; supervision; project administration; writing – review & editing.

## Conflicts of interest

There are no conflicts to declare.

## Supplementary Material

SC-017-D6SC06134F-s001

## Data Availability

All study data are included in the article and supplementary information (SI). Supplementary information: synthetic procedures, ion exchange and membrane fabrication protocols, PXRD patterns, N_2_ adsorption isotherms, FTIR spectra, XPS analysis, SEM-EDS mapping, ICP-OES calibration data, electrochemical impedance spectroscopy data, transference number measurements, and additional electrochemical testing results. See DOI: https://doi.org/10.1039/d6sc06134f.
