# Supplementary material for: Intrinsic linker flexibility in an anionic metal–organic framework electrolyte for selective lithium-ion conduction
Source: Chem Sci. 2026 Aug 28;17(40):19359–63. doi: 10.1039/d6sc06134f (PMC13523094; doi:10.1039/d6sc06134f)
Supplement: Supplementary file 1 [file SC-017-D6SC06134F-s001.pdf]

Supplementary Information (SI) for Chemical Science.
This journal is © The Royal Society of Chemistry 2026

SUPPORTING INFORMATION

Intrinsic Linker Flexibility in an Anionic Metal-Organic Framework Electrolyte for Selective Lithium-Ion Conduction

Zina Deriche[a], Vallabha Rao Rikka[d], Isaac Metcalf[a], Ahad Hussain Javed[a], Satya Prakash Suman[c], Moneer Alenezi[a], Aditya Mohite[a,b], Sibani Lisa Biswal[*,a,b], and Stavroula Kampouri[*,a,b,c]

[a] Department of Chemical and Biomolecular Engineering, Rice University, Houston, TX 77005
[b] Rice Advanced Materials Institute (RAMI), Rice University, Houston, TX 77005
[c] Chemistry Department, Rice University, Houston, TX 77005
[d] Electrochemical Safety Research Institute (ESRI), Houston, TX 77023

1. Experimental Section

1.1 Materials

$Zn(NO_3)_2 \cdot 6H_2O$ (98%), 1,2,3,4-butanetetracarboxylic acid (BTCA, 99%), NaOH (≥97%), polyethylene oxide (PEO, Mw ≈ 600k), lithium bis(trifluoromethanesulfonyl)imide (LiTFSI, 99.99%), aluminium sulphate 18 hydrate ($Al_2(SO_4)_3 \cdot 18H_2O$) and fumaric acid, and anhydrous acetonitrile (99.8%) were purchased from Sigma-Aldrich and used without further purification. Lithium metal foils (16 mm diameter, 0.6 mm thick), stainless steel CR2032 coin-cell components, and conductive carbon black were obtained from MSE Supplies.

1.2 Synthesis

ZnBTCA was synthesized via a two-step precipitation–coordination approach in aqueous media, adapted from previously reported procedures.[1] $Zn(NO_3)_2 \cdot 6H_2O$ (0.136 g, 0.46 mmol) was first dissolved in 5 mL of deionized water. To this solution, 1 mL of 1 M NaOH was added. Subsequently, a BTCA solution (0.127 g, 0.54 mmol) prepared in 10 mL of deionized water was added, and the resulting mixture was sonicated for 10 minutes. The suspension gradually dissolved into a clear solution, which was then slowly evaporated at 70 °C for 48 hours, yielding colorless single crystals. The product was washed by centrifugation with deionized water, followed by solvent exchange with methanol and vacuum drying at 120 °C. Crystalline ZnBTCA was obtained in approximately 67% yield.

Al-fumarate was synthesized under hydrothermal conditions using aluminum sulfate $Al_2(SO_4)_3 \cdot 18H_2O$, fumaric acid, and water. For a 23 mL reaction, aluminum sulfate octadecahydrate (1.18 g, 1.77 mmol) was dissolved in water. Fumaric acid (0.41 g, 3.54 mmol) was then added, corresponding to a 1:1 fumaric acid-to-Al molar ratio. The mixture was transferred to a Teflon-lined autoclave and heated at 110 °C for 32 h. The resulting solid was collected and washed thoroughly with deionized water, followed by solvent exchange with methanol and vacuum drying at 120 °C.[14]

1.3 Ion Exchange and Membrane Fabrication

ZnBTCA crystals were soaked in 1 M LiTFSI in acetonitrile for 24 h inside an argon glovebox, filtered, and vacuum-dried at 100 °C. The crystals were ground with 5 wt% PEO in acetonitrile until a dough-like consistency formed, then pressed at 1 MPa into free-standing membranes (0.2–0.5 mm thick)

1.4 Characterizations

Powder X-ray diffraction (XRD) patterns were recorded on the D6 Phaser Bruker benchtop XRD (Cu Kα Radiation, λ = 1.54 Å). Inductively coupled plasma optical emission spectroscopy (ICP-OES) was conducted on Perkin Elmer Avio 550 ICP-OES. Indentation studies and fitting were all completed on the Hysitron TI 980 TriboIndenter. X-ray photoelectron spectroscopy (XPS) was conducted on a Thermo Scientific Nexsa G2. FT-IR spectra were recorded on the Thermo Scientific Nicolet. Scanning electron microscope (SEM) images were collected from JEOL IT800 with JEOL Energy Dispersive X-ray Spectrometer (EDS).

1.5 Electrochemical impedance spectroscopy (EIS)

Pellets were prepared in a home-built Swagelok-type cell by pressing the powder between stainless-steel (SS) blocking electrodes (1.23 cm diameter) under a dry N_2 atmosphere. Pressure was applied by tightening the cell hardware. The resulting pellet thicknesses were typically 0.2–0.5 mm. AC impedance was measured in a two-electrode configuration (Bio-Logic SP-150e) over 1 MHz–1 Hz with a 100 mV AC perturbation. Temperature was controlled in an oven; at each set point (20–100 °C in 20 °C increments), the sample was equilibrated for 1 h prior to acquisition. Nyquist spectra were fit in EC-Lab to the equivalent circuit shown in Circuit S1 to extract the resistance R. Ionic conductivity was then calculated using:

$$\sigma = \frac{L}{RA} \quad (1)$$

where L is the pellet thickness, and A is the electrode contact area.[1-4] Activation energy (Ea) was obtained by fitting the temperature dependence of the ionic conductivity to an Arrhenius form (consistent with the Nernst–Einstein framework):

$$\sigma\ (T) = \sigma_0 \exp\left(-\frac{E_a}{k_B T}\right) \quad (2)$$

where σ_0 is the pre-exponential factor, T is the absolute temperature, and k_B is Boltzmann's constant. E_a was extracted from the slope of ln σ vs 1000/T.

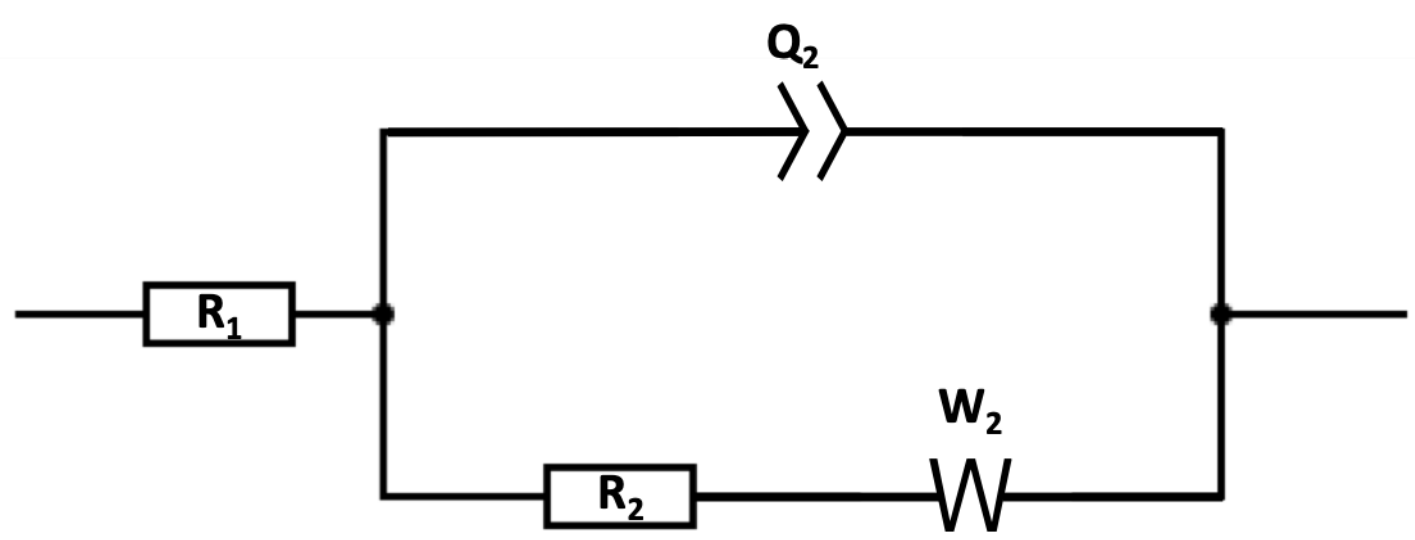

Circuit S1 Equivalent circuit model used to fit the electrochemical impedance spectroscopy (EIS) Nyquist plots. The circuit consists of a series resistance (R_1) connected to a parallel combination of a constant phase element (Q_2) and a branch containing the resistance R_2 and Warburg diffusion element W_2. The Warburg element accounts for diffusion-controlled impedance.

1.6 Transference Number Measurements

The Li^+ transference number (t_{Li+}) was determined by the chronoamperometry test using symmetric lithium cells at an applied DC voltage of 50 mV.[4,5] EIS was also performed before and after the DC polarization with the frequency ranging from 1 MHz to 1 Hz. The t_{Li+} value was calculated by Evans-Vincent-Bruce's equation:

$$t_{Li^+} = \frac{I_{ss}(V - I_0 R_0)}{I_0(V - I_{ss} R_{ss})} \quad (3)$$

Where V is the polarization voltage, I_0 is the initial current, I_{ss} is the steady state current, R_0 is the initial total resistance, and R_{ss} is the steady state total resistance. R_0 and R_{ss} were obtained by fitting the Nyquist plot in Fig. S11 to Circuit S2 using EC-Lab.

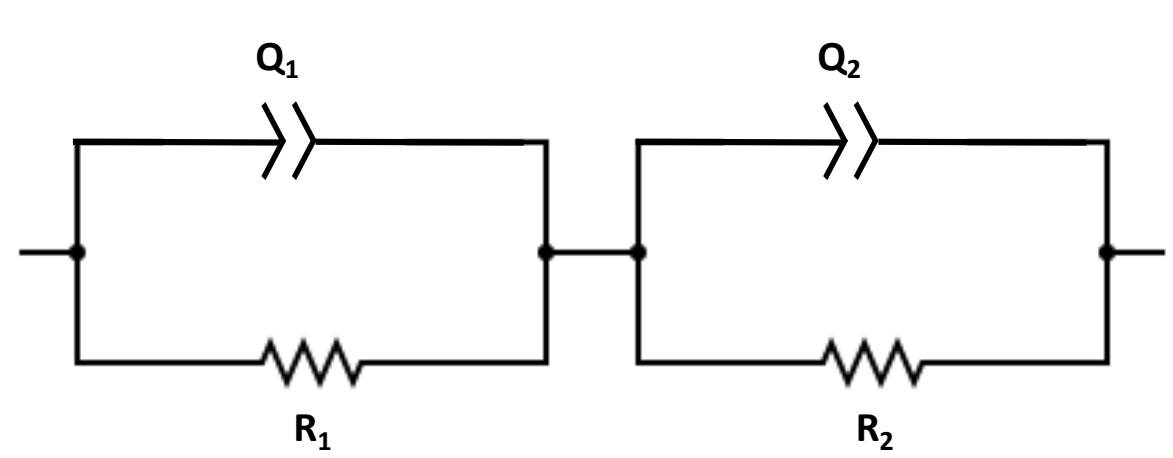

Circuit S2 This circuit is used for fitting Nyquist plots for symmetric cells. Q_1 and Q_2 are constant phase elements, and R_1 and R_2 are the resistors.

1.7 Cell Assembly and Electrochemical Testing

Symmetric Li|ZnBTCA|Li cells were assembled in CR2032 coin cells with ZnBTCA as either a pellet or a membrane sandwiched between two Li foils. Cells were sealed in an argon-filled glove box. The symmetric cell, Li|ZnBTCA@5-PEO|Li, was periodically charged and discharged for 1 hour at different current densities of 0.2, 0.5, and 1 mA cm−2 at room temperature using an Arbin cell tester.

1.8 ICP-OES Calculations

Using the ICP-OES results summarized in Table S4, the Li, Na, and Zn contents were converted into molar ratios in order to determine the extent of Li^+ incorporation and decrease in residual Na^+ content after ion exchange. These values were then used to calculate the percentage of Li^+ among the mobile charge-balancing cations, as summarized in Tables S5 and S6.

First, the amount of each element was calculated from the measured ICP-OES concentration:

$$n_i = \frac{C_i V}{M_i} \quad (4)$$

where i corresponds to Zn, Na, or Li; C_i is the ICP-OES concentration of element i in mg L^{-1}, V is the final solution volume in L, and M_i is the molar mass of the element in mg $mmol^{-1}$.

The Na^+ and Li^+ contents were then normalized to two Zn atoms, consistent with the empirical ZnBTCA framework formula:

$$Na\ per\ 2\ Zn = 2 \times \frac{n_{Na}}{n_{Zn}} \quad (5)$$

$$Li\ per\ 2\ Zn = 2 \times \frac{n_{Li}}{n_{Zn}} \quad (6)$$

The exchanged material was therefore expressed using the general empirical formula:

$$\left[Zn_2(BTCA)(H_2O)_{4-(x+y)}(OH)_{x+y}\right] \cdot Na_xLi_y \qquad (7)$$

where x is the Na content per two Zn atoms and y is the Li content per two Zn atoms determined from ICP-OES.

The percentage of Li^+ among the mobile charge-balancing cations was calculated as:

$$\%\ mobile\ Li = \frac{n_{Li}}{n_{Li} + n_{Na}} \times 100 \qquad (8)$$

Equivalently, this can be calculated using the normalized values:

$$\%\ mobile\ Li = \frac{y}{x + y} \times 100 \qquad (9)$$

where x and y are the Na and Li contents per two Zn atoms, respectively.

1.9 Indentation Calculations

Nanoindentation data were analyzed using the Oliver–Pharr method. The reduced modulus (E_r) and hardness were obtained directly from the Hysitron TriboIndenter analysis software based on the unloading stiffness and projected contact area. The Young's modulus of the sample (E_s) was then calculated from the reduced modulus according to:

$$\frac{1}{E_r} = \frac{1 - v_s^2}{E_s} + \frac{1 - v_i^2}{E_i} (10)$$

where E_i = 1141 GPa which is the Young's modulus of the indenter, ν_I = 0.07 is the Poisson's ratio of the indenter, and ν_s = 0.30 is the Poisson's ratio of the sample.

2. Results

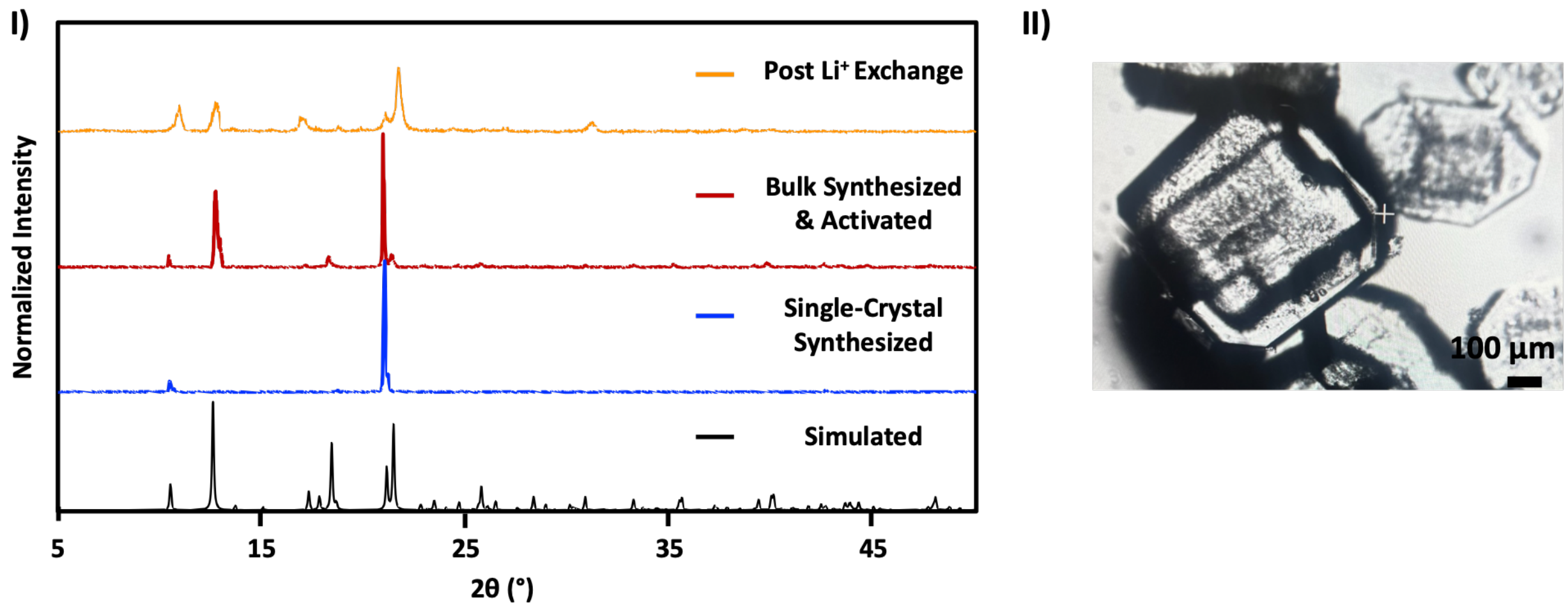

Fig. S1 (I) PXRD patterns of simulated ZnBTCA (black), as-synthesized single crystals (blue), bulk-synthesized ZnBTCA after vacuum activation (red), and ZnBTCA after Na^+/Li^+ ion exchange (orange), confirming phase purity and retention of the framework after activation and ion exchange. (II) Optical micrograph showing well-faceted ZnBTCA crystals.

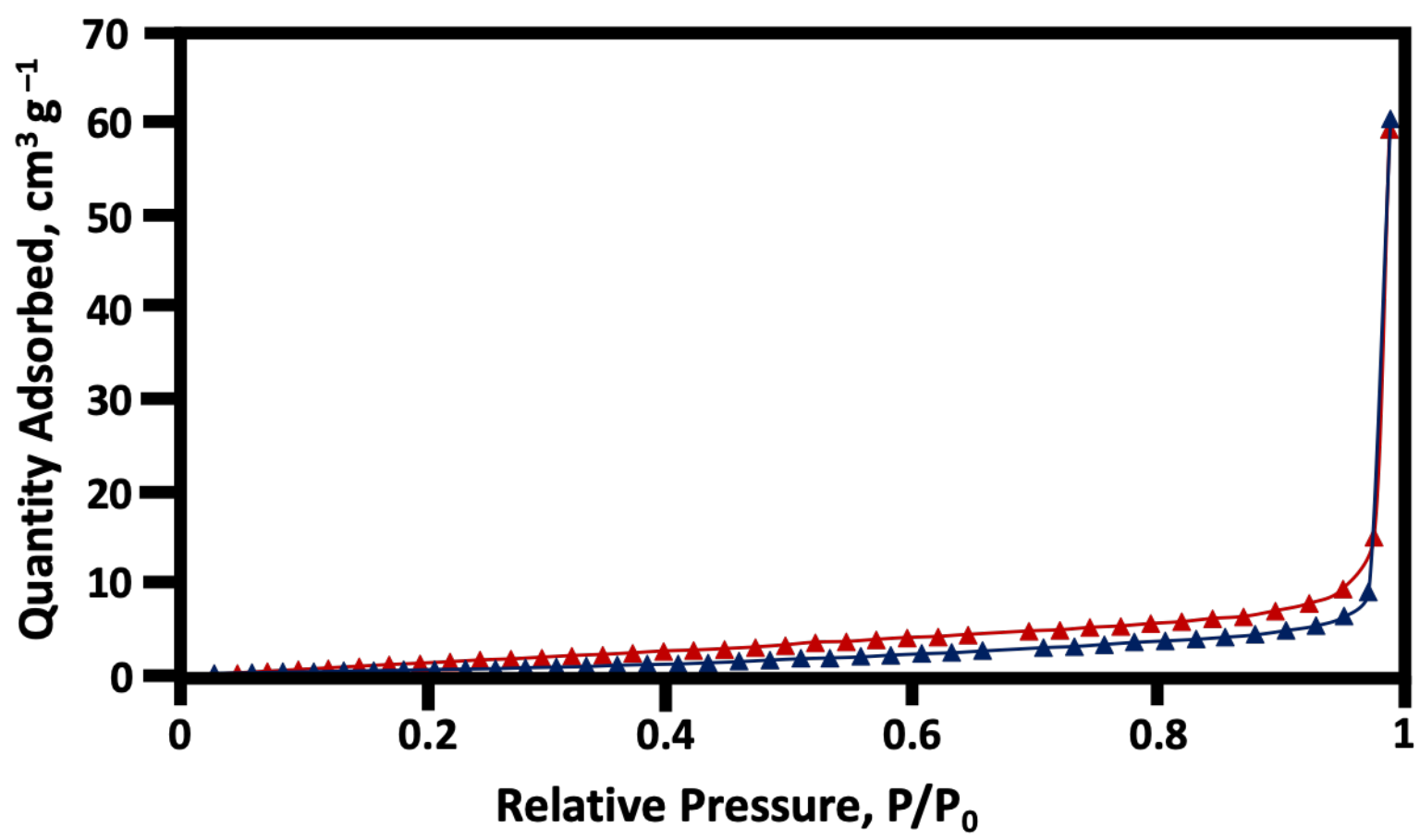

Fig. S2 N_2 adsorption–desorption isotherms of ZnBTCA measured at 77 K. The isotherm exhibits Type III-like behavior, with limited uptake over most of the relative-pressure range and a sharp increase near $P/P_0=1$, consistent with weak N_2–framework interactions and condensation within interparticle voids. The limited N_2 uptake indicates restricted accessibility under the measurement conditions rather than an absence of crystallographically defined porosity. A Mercury calculation on the guest-free SC-XRD structure gives a theoretical N_2-accessible surface area of 315 $m^2\ g^{-1}$ using a probe radius of 1.86 Å.

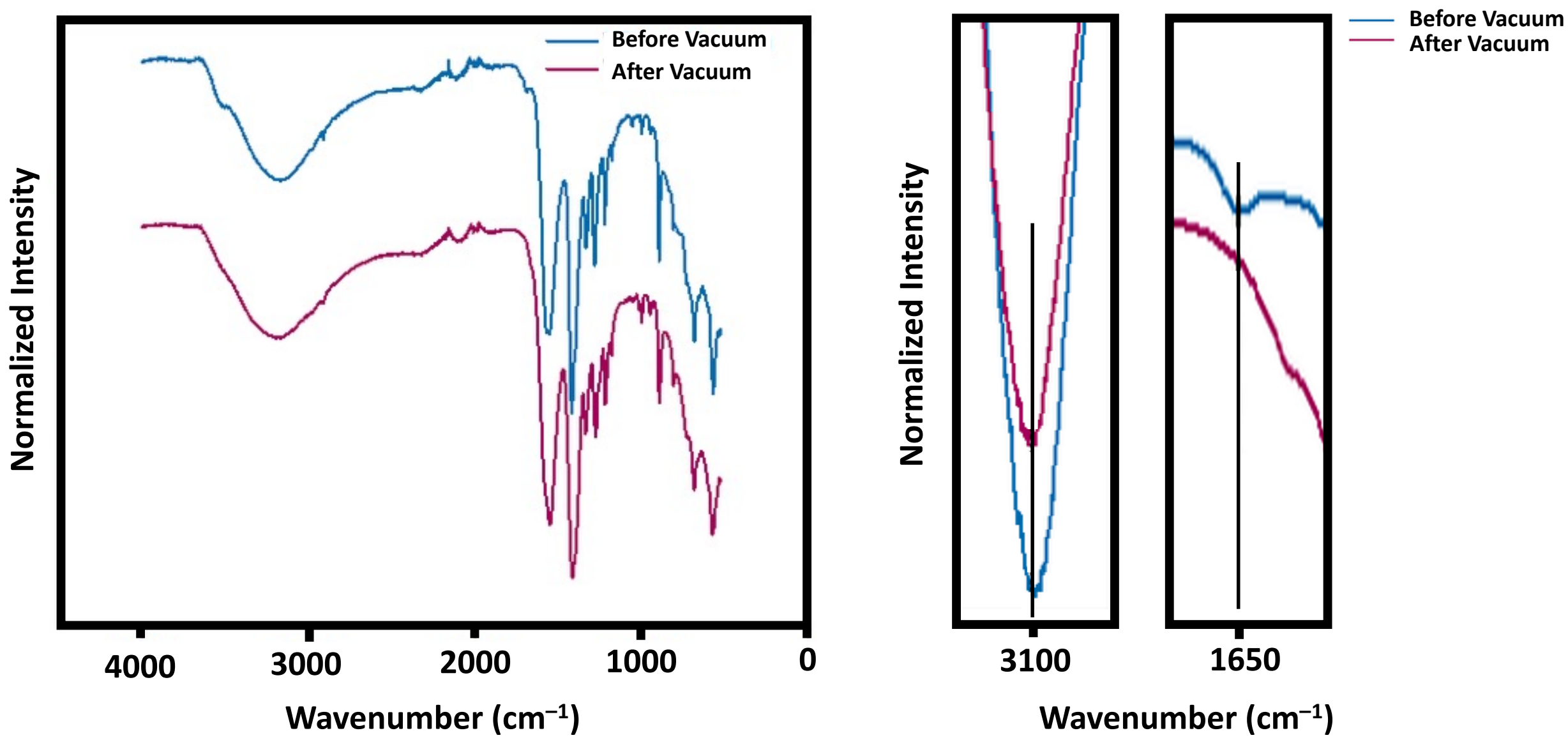

Fig. S3 Fourier-transform infrared (FTIR) spectra of ZnBTCA collected before (blue) and after (maroon) thermal activation at 120 °C under vacuum. The decrease in the broad O–H stretching band at approximately 3100 cm^{-1} and the H–O–H bending vibration at approximately 1650 cm^{-1} is interpreted qualitatively as evidence of reduced water content. A residual feature in the O–H region remains, consistent with coordinated water or hydroxyl groups associated with the framework.

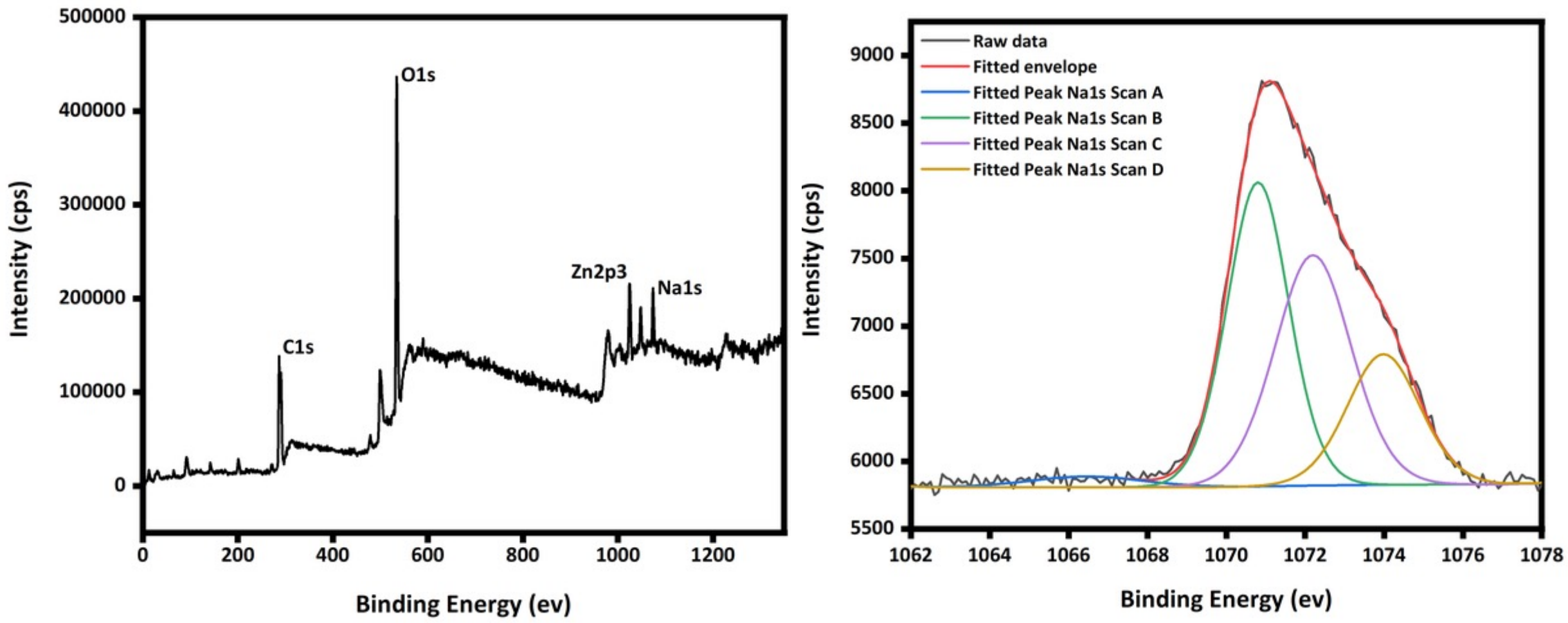

Fig. S4 X-ray photoelectron spectroscopy (XPS) of ZnBTCA. (Left) Survey spectrum showing C 1s (~285 eV), O 1s (~532 eV), Zn 2p (≈1020–1045 eV), and a clear Na 1s signal (~1071 eV). (Right) High-resolution Na 1s region with Shirley background subtraction and pseudo-Voigt fits for four independent scans (A–D); the summed fit (red) overlays the raw data, confirming a reproducible Na 1s peak at 1071–1073 eV. Binding energies were referenced to C 1s = 284.8 eV. The persistent Na signal corroborates an anionic framework charge-balanced by extra framework Na^+.

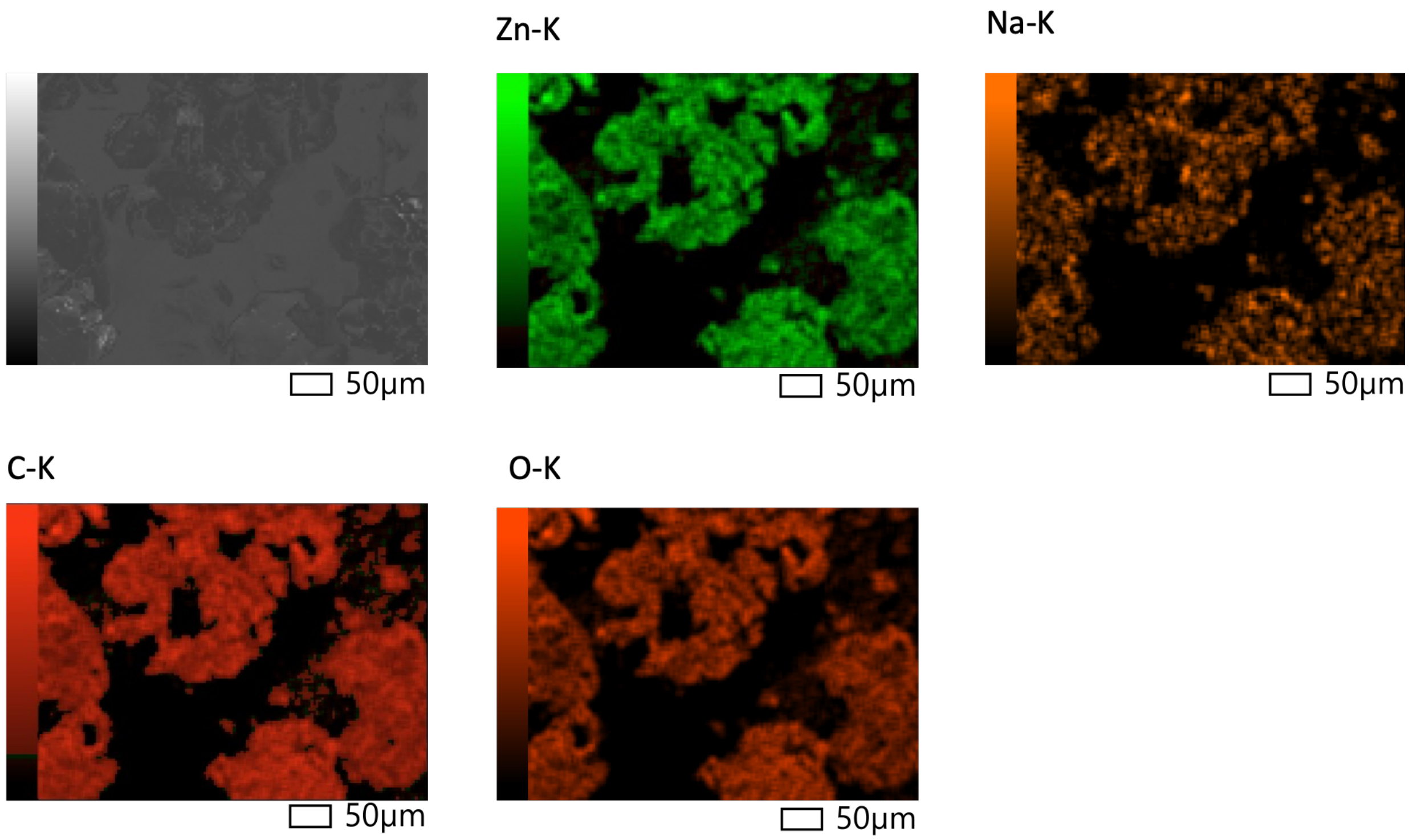

Fig. S5 SEM–EDS elemental mapping of ZnBTCA particles. The grayscale panel shows the SEM reference image; colored panels are EDS Kα maps for Zn, Na, C, and O (intensity increases with color scale at left of each map). Zn, C, and O signals delineate the MOF particles, and the Na map is largely co-localized with Zn/O within the same regions, with negligible signal in the surrounding voids—consistent with Na^+ residing in the framework rather than the substrate.

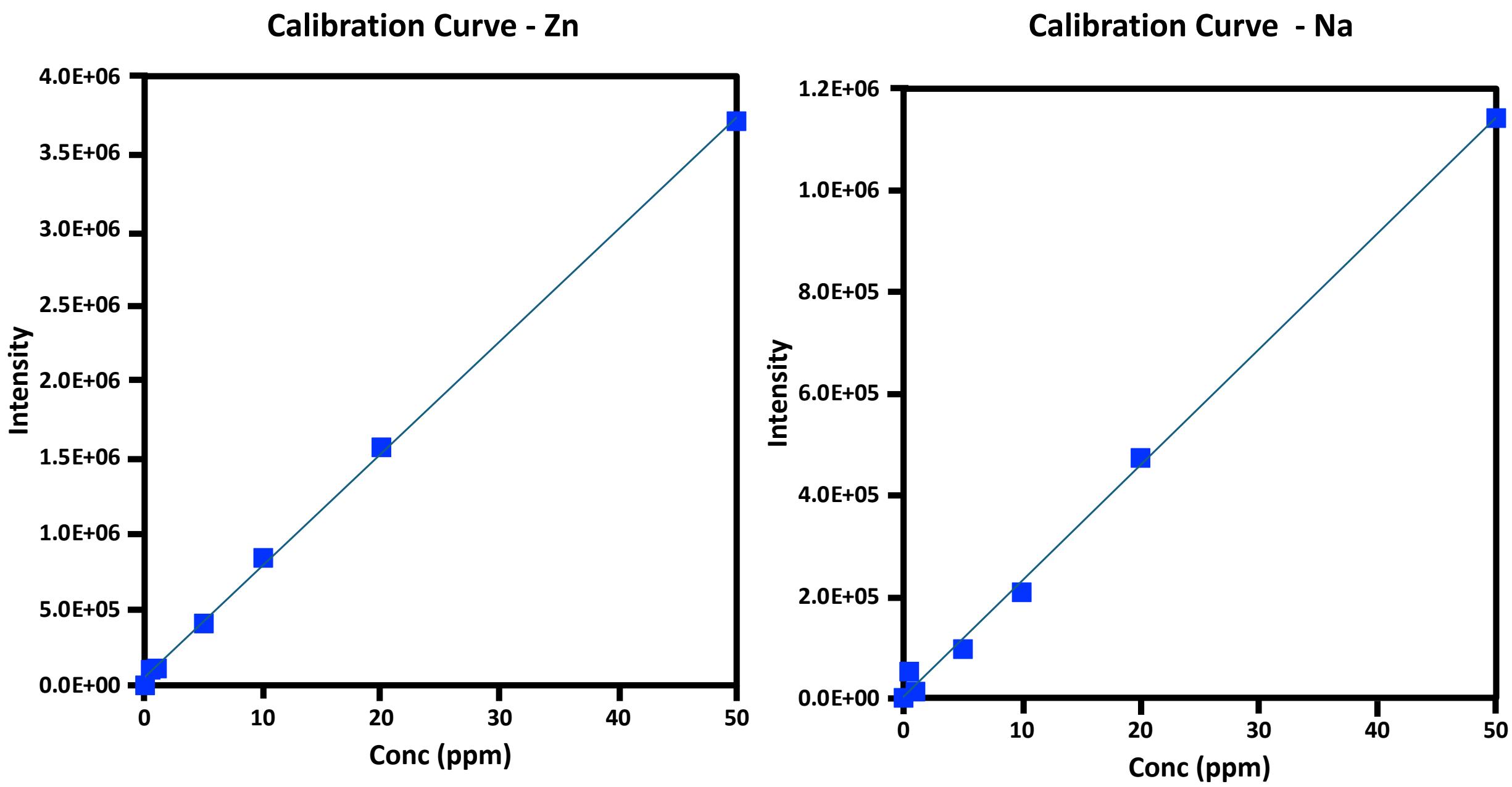

Fig. S6 ICP-OES calibration curves for Zn and Na used to quantify cation content in digested ZnBTCA samples. Matrix-matched standards (0–50 ppm in 2% HNO_3) were measured in triplicate; mean intensities are plotted with linear fits used for quantitation (error bars smaller than symbols).

Table S1. Raw ICP-OES Data Concentrations

Sample Id	Zn (mg/L)	Na (mg/L)
ZnBTCA	112.395	50.106
RSD	*0.7%*	*0.6%*
Replicate1	112.451	50.192
Replicate2	111.533	49.766
Replicate3	113.201	50.359

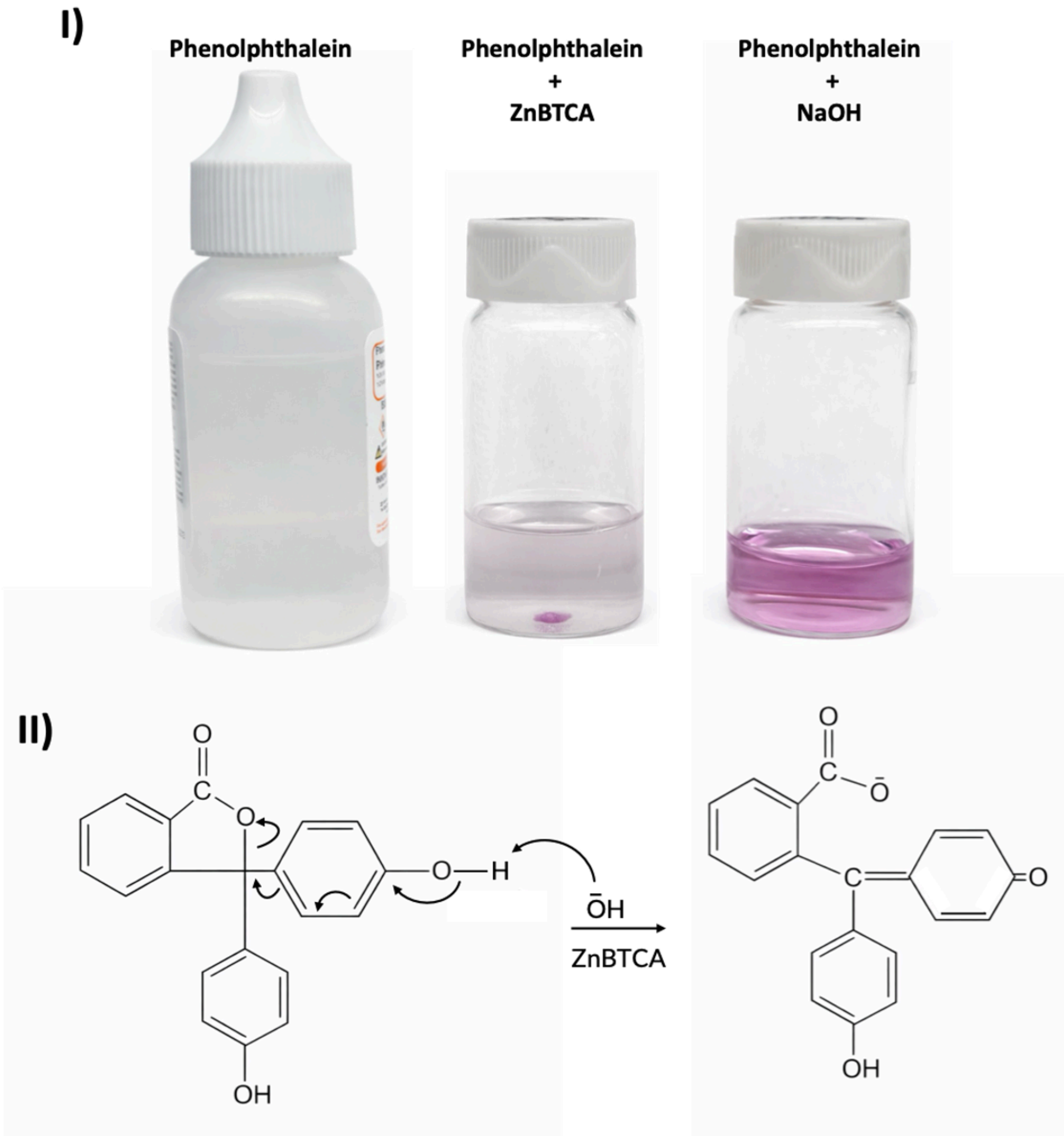

Fig. S7 (I) Photographs of phenolphthalein solution alone (colorless), phenolphthalein in the presence of Zn-BTCA, and phenolphthalein with NaOH (positive control). The development of a light pink coloration upon addition of Zn-BTCA indicates partial deprotonation of phenolphthalein, consistent with the presence of accessible basic hydroxide (OH^-) sites associated with the framework. The stronger pink color observed with NaOH confirms the expected OH^- response. (II) Schematic illustrates the base-induced ring opening of phenolphthalein to its conjugated quinonoid form, responsible for the pink color.

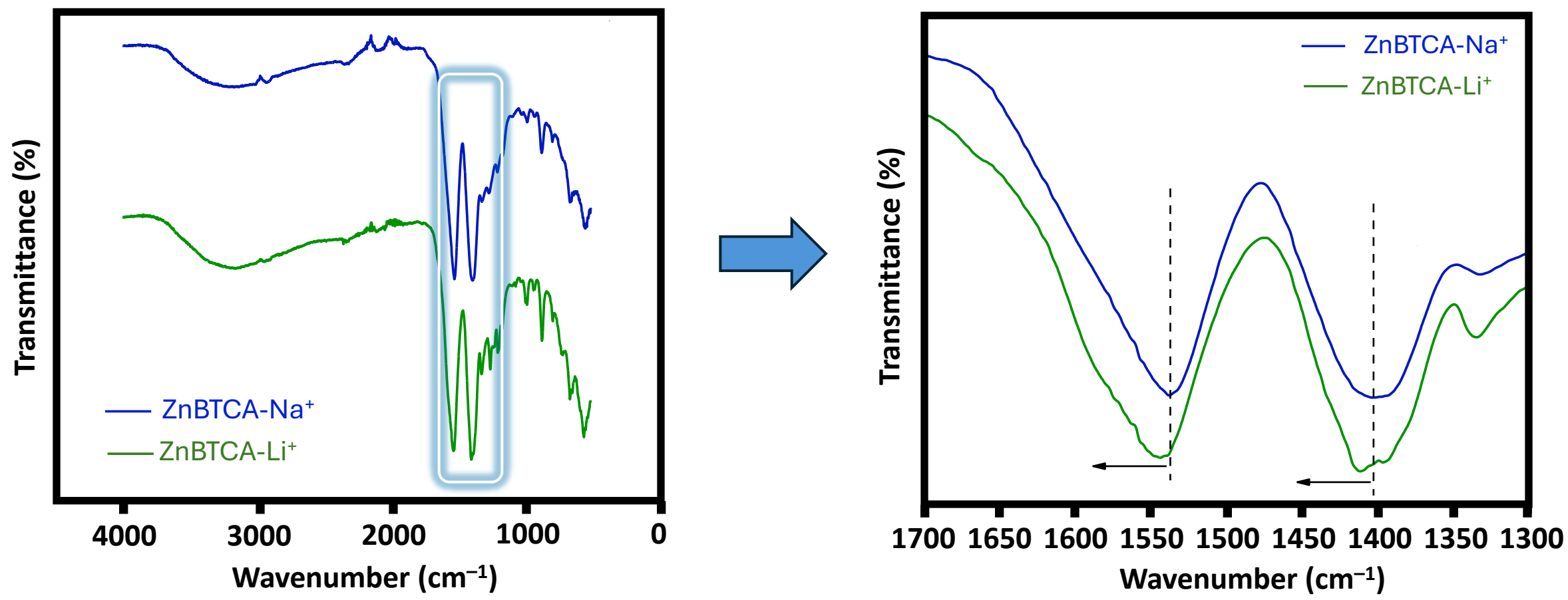

Fig. S8 Fourier-transform infrared (FTIR) spectra comparing ZnBTCA synthesized in the presence of Na^+ (blue) versus after Li^+ exchange (green). (Left) Full-range FTIR spectra highlighting differences in the fingerprint region. (Right) Magnified view of the carboxylate stretching region (1300–1700 cm^{-1}), showing a red-shift in both the asymmetric (~1550 cm^{-1}) and symmetric (~1400 cm^{-1}) –COO– stretching bands upon Li^+ incorporation. These shifts are attributed to stronger coordination between the more polarizing Li^+ ion and the carboxylate groups, indicative of successful cation exchange and altered electronic environments within the framework.

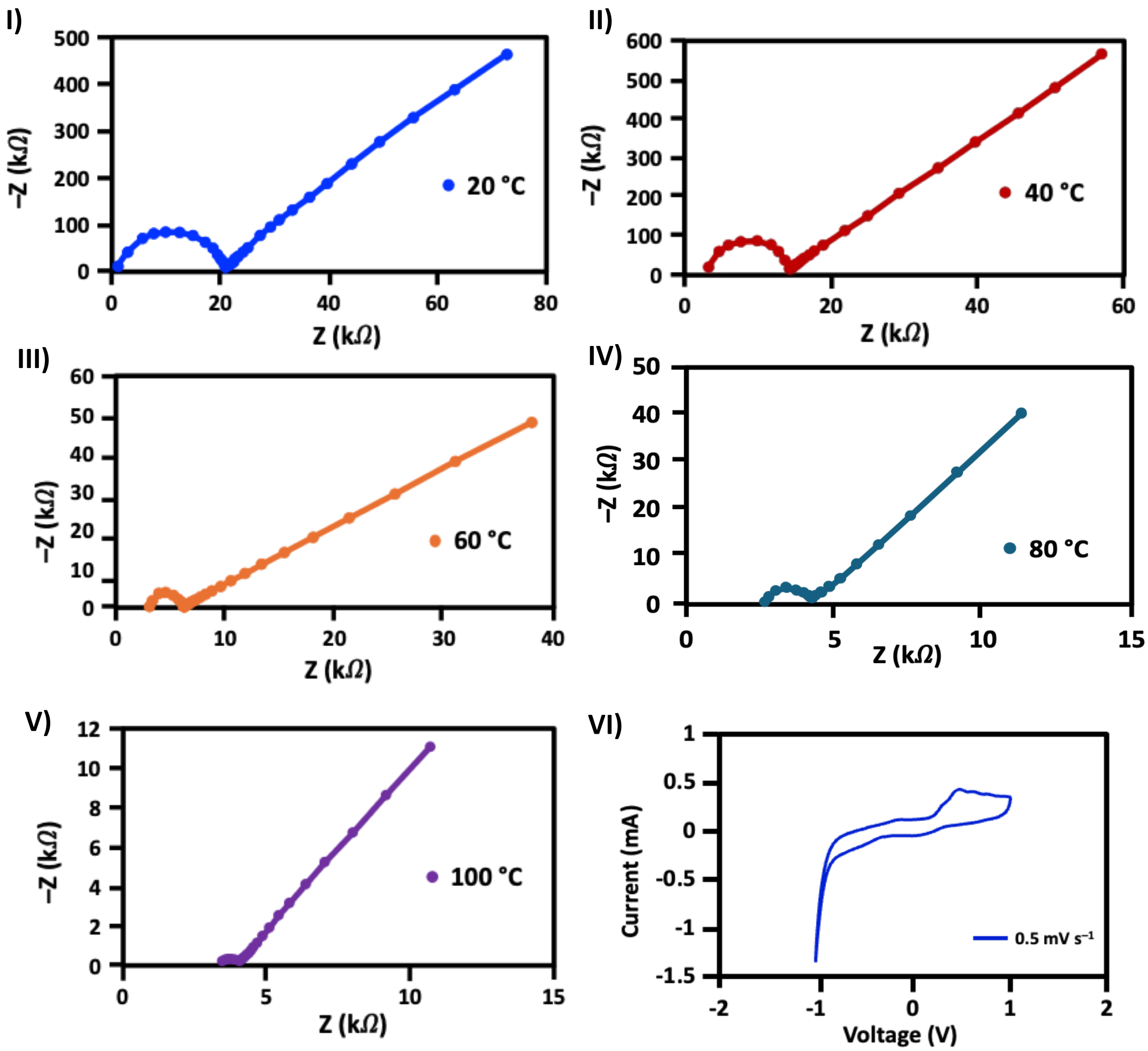

Fig. S9 Temperature-dependent impedance spectra and cyclic voltammogram of ZnBTCA. Nyquist plots of ZnBTCA measured using stainless-steel blocking electrodes at (I) 20 °C, (II) 40 °C, (III) 60 °C, (IV) 80 °C, and (V) 100 °C. All impedance spectra were fitted using equivalent circuit S1, and the extracted fitting parameters are summarized in Table S7. (VI) Cyclic voltammogram of the Li|ZnBTCA|Li symmetric cell collected at a scan rate of 0.5 mV s^{-1}.

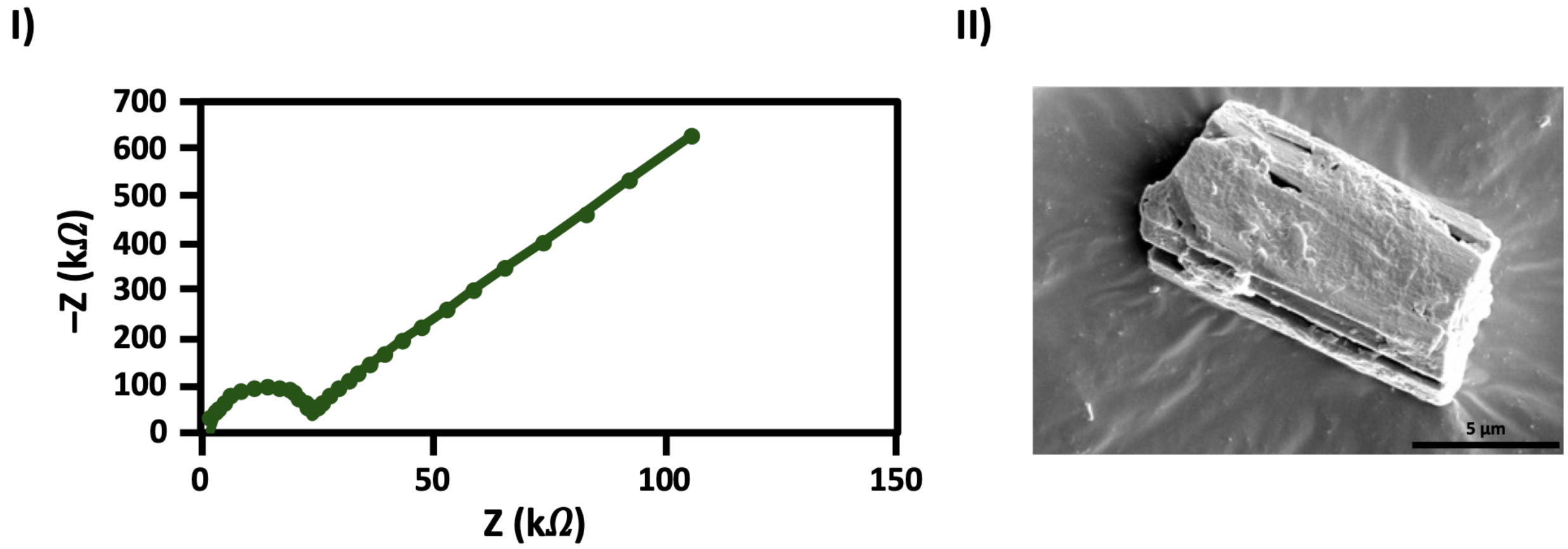

Fig. S10 Effect of particle-size reduction on ZnBTCA ionic conductivity. (I) Nyquist plot of size-reduced ZnBTCA crystals measured at 20 °C using stainless-steel blocking electrodes. The extracted room-temperature effective ionic conductivity is 2.33 x 10^{-5} S cm^{-1}. The $R_{electrolyte}$ is 1819.2, A is 1.18 cm^2, and l is 0.05 cm. (II) SEM image of the size-reduced ZnBTCA crystal, showing an approximate particle size of ~11 µm. The impedance spectrum was fitted using equivalent circuit S1.

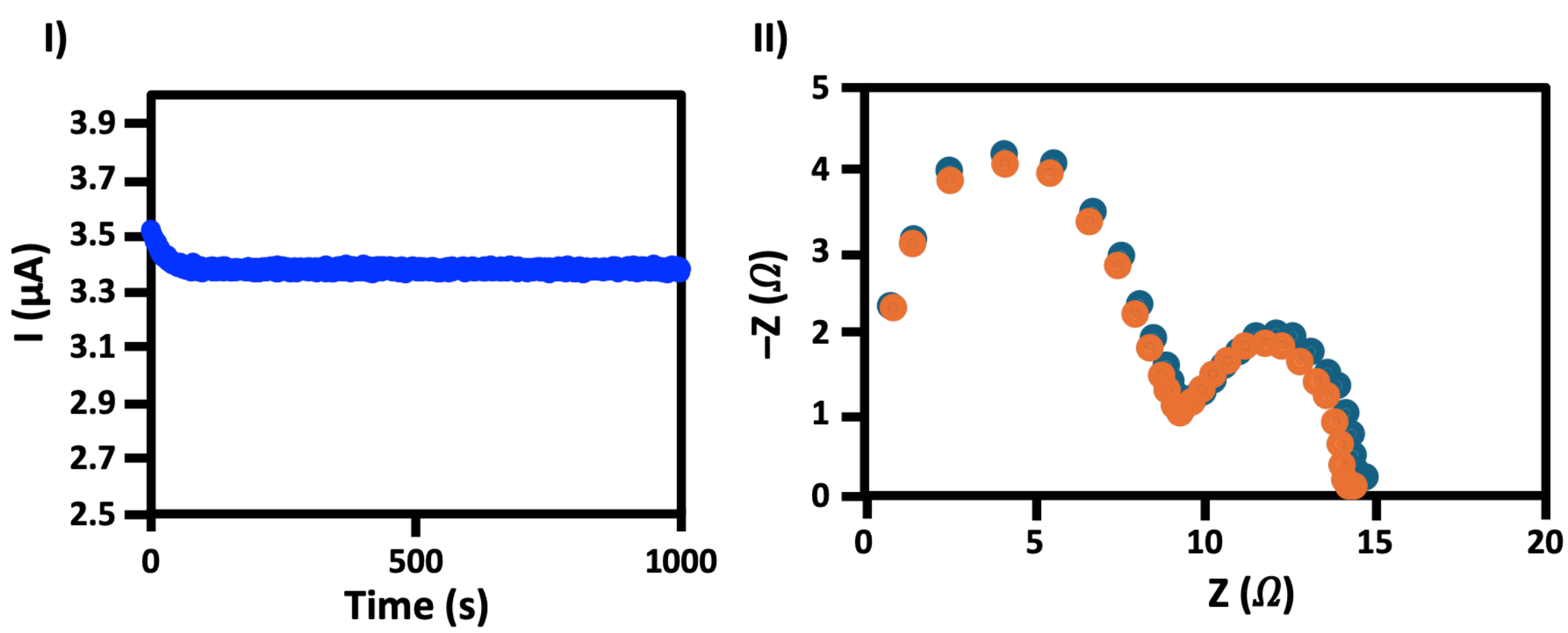

Fig. S11 (I) Shows a chronoamperogram during the polarization of a Li |ZnBTCA| Li cell at room temperature, with an applied potential of 50 mV. (II) Nyquist plot of the Li | ZnBTCA| Li cell at room temperature before and after the polarization experiment. Values obtained from each plot: I_0 = 3.52 µA, I_{ss} = 3.311 µA, R_0 = 9461 Ω, R_{ss} = 9004 Ω

Table S2. Reported Young's Modulus Values for MOFs

MOF Type	Young Modulus (GPa)
UiO-66(Zr)[10]	22.0-45.0
NU-1000-bip[11]	20
DUT-47(lp)[12]	11.1
MOF-808(Zr)[13]	26.9
MOF-5[10]	2.7
ZnBTCA	**4.6**

Table S3. Reported Li^+ Transference Number

MOF Type	T_{Li+}
UiO-66(Zr)[6]	0.9
MIT-20-LiCl[4]	0.66
InOF[7]	0.78
NiCo-MOF[9]	0.49
Co-MOF[8]	0.41
ZnBTCA	**0.79**

Table S4. Raw ICP-OES Data for Different Li Soaking Times and Different Li Concentrations for 24 hrs

Sample Id	Zn (mg/L)	Zn (RSD %)	Na (mg/L)	Na (RSD %)	Li (mg/L)	Li (RSD %)
0 hr Li	23.476	0.9%	10.611	0.9%	0.001	0.6%
0.5 hr Li	53.542	0.7%	7.986	0.6%	2.220	0.9%
1 hr Li	39.469	0.5%	4.409	0.4%	2.739	0.9%
8 hr Li	32.979	0.9%	3.423	0.7%	4.211	0.5%
12 hr Li	45.985	0.8%	2.468	0.7%	6.115	0.7%
24 hr Li	40.681	0.6%	1.039	0.7%	5.507	0.8%
48 hr Li	37.720	0.6%	1.020	0.6%	5.516	0.5%
0.5 M Li	27.012	0.6%	4.403	0.3%	3.190	0.8%
1 M Li	28.897	0.9%	0.809	0.9%	4.062	0.6%
1.5 M Li	36.266	0.2%	1.007	0.2%	4.928	0.8%
2 M Li	28.604	0.5%	0.819	0.3%	4.567	0.8%
3 M Li	30.657	0.8%	0.709	0.6%	5.065	0.8%

Table S5. Time-dependent Li^+ incorporation and decrease in residual Na^+ in ZnBTCA after soaking in 1 M LiTFSI. The Na and Li contents are normalized per two Zn atoms, where x and y correspond to the empirical formula in equation 7 in the SI.

Soaking Time (hr) in 1M Li	Na (x)	Li (y)	% Li of Mobile Cations
0	2.57	0.00	0.00
0.5	0.85	0.78	47.94
1	0.64	1.31	67.29
8	0.59	2.41	80.30
12	0.31	2.51	89.14
24	0.15	2.55	94.61
48	0.15	2.76	94.71

Table S6. ICP-data for soaking of ZnBTCA at different Li concentrations for 24 hrs. The Na and Li contents are normalized per two Zn atoms, where x and y correspond to the empirical formula in equation 7 in the SI.

Concentrations of Li for 24 hr (M)	Na (x)	Li (y)	% Li of Mobile Cations
0.5	0.93	2.23	70.59
1	0.16	2.65	94.33
1.5	0.16	2.56	94.19
2	0.16	3.01	94.86
3	0.13	3.11	95.95

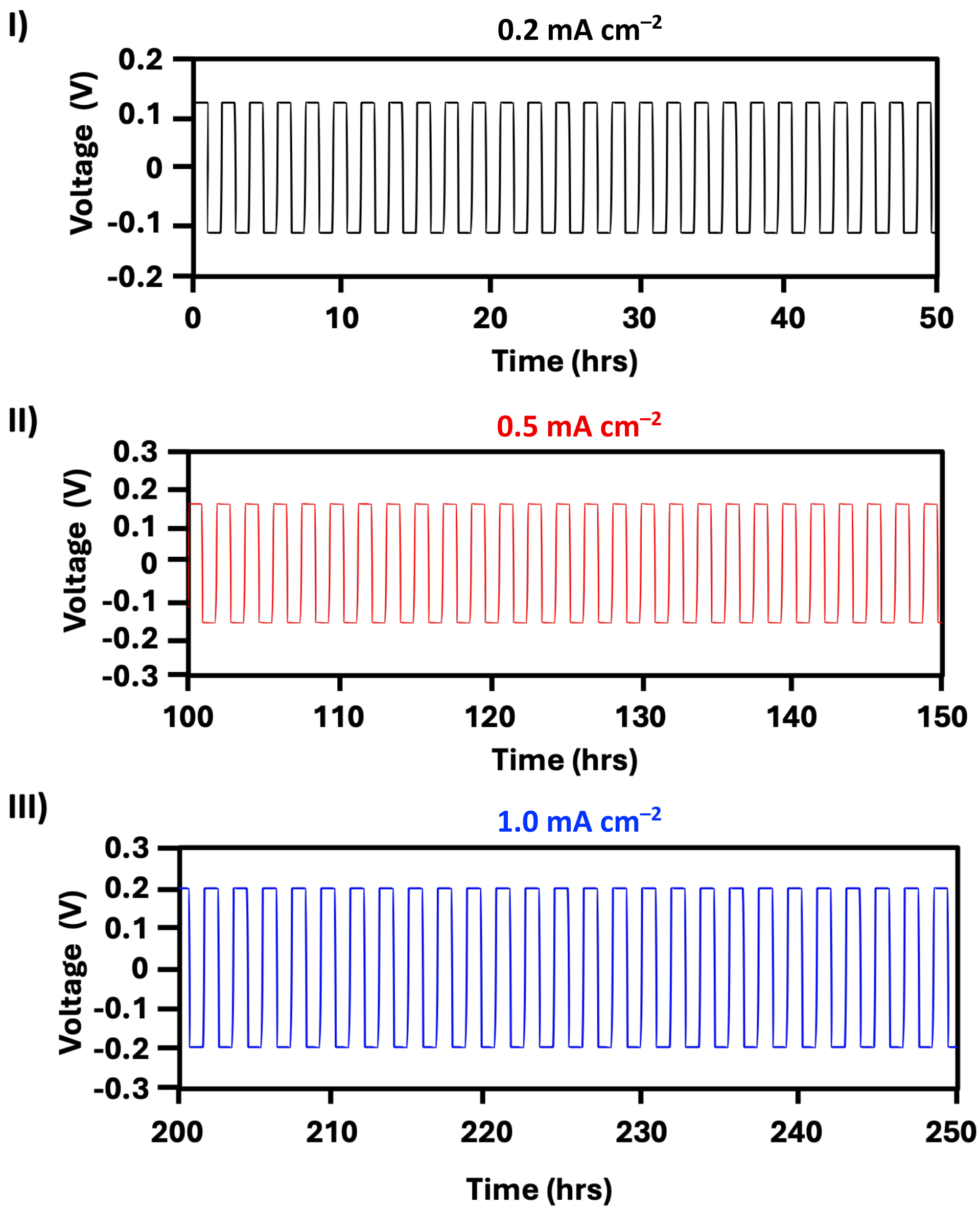

Fig. S12 Zoomed-in voltage profiles of Li|ZnBTCA@5-PEO|Li symmetric-cell cycling. Representative zoomed-in regions of the galvanostatic cycling profile at current densities of (I) 0.2 mA cm^{-2}, (II) 0.5 mA cm^{-2}, and (III) 1.0 mA cm^{-2}. The zoomed-in profiles show stable voltage polarization during Li plating/stripping at each current density.

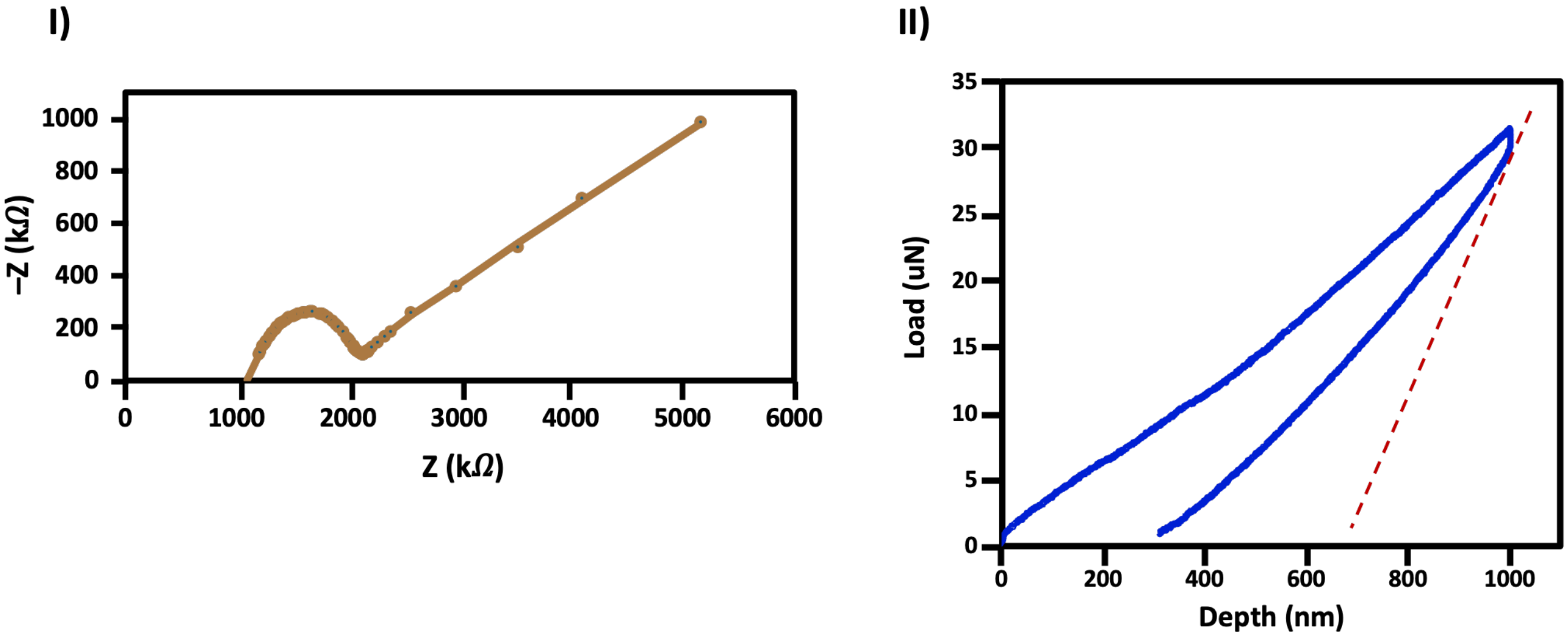

Fig. S13 Room-temperature ionic conductivity and mechanical response of Al-fumarate, included as a non-aromatic carboxylate MOF reference. (I) Nyquist plot measured using stainless-steel blocking electrodes, yielding an ionic conductivity of 1.42×10^{-6} S cm^{-1}. The $R_{electrolyte}$ is 29840, A is 1.18 cm^{2}, and I is 0.05 cm. (II) Nanoindentation load–displacement curve of Al-fumarate, from which the Young's modulus was experimentally determined to be 5.7 GPa. The corresponding reduced modulus (E_r) was 6.24 GPa.

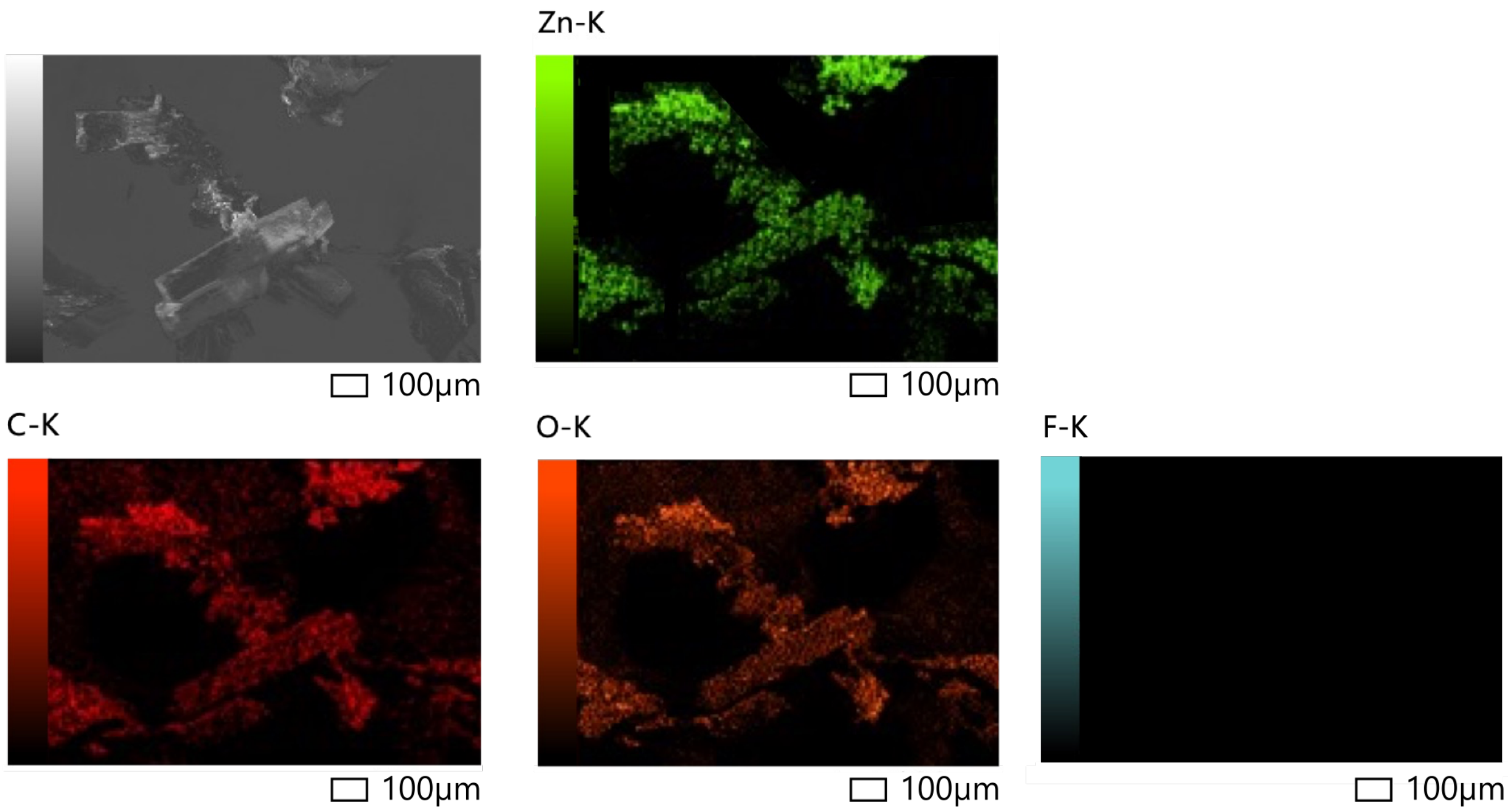

Fig. S14 SEM–EDS elemental mapping of ZnBTCA particles after lithiation. The grayscale panel shows the SEM reference image, while the colored panels display the corresponding EDS Kα maps for Zn, C, O, and F. Zn, C, and O are distributed throughout the particles, whereas no detectable fluorine signal is observed.

Table S7. Equivalent-circuit fitting parameters and ionic-conductivity calculations for ZnBTCA. R_2 is the electrolyte resistance, a_2 is the dimensionless constant-phase-element exponent, and s_2 is the Warburg coefficient. A and l are the pellet–electrode contact area and pellet thickness. The ionic conductivity, σ_i, was calculated using Equation S1.

Temperature (°C)	R_2=$R_{electrolyte}$ (Ω)	a_2	s_2 (Ω $s^{-1/2}$)	A (cm^2)	l (cm)	σ_i (S cm^{-1})
20	1203.21	0.8472	42720	1.18	0.05	3.53×10^{-5}
40	658.13	0.7033	29710	1.18	0.05	6.44×10^{-5}
60	272.32	0.7524	13183	1.18	0.06	1.87×10^{-4}
80	134.2	0.8471	6508	1.18	0.06	3.79×10^{-4}
100	65.61	0.9422	5506	1.18	0.05	6.46×10^{-4}

3 References

1 Z. Deriche, S. L. Biswal and S. Kampouri, *J. Mater. Chem. A*, 2025, **13**, 33012–33033.

2 P. Shewmon *Diffusion in Solids* , Springer International Publishing, Cham s.l, 2016.

3 R. A. Kharod , J. L. Andrews and M. Dincă , *Annu. Rev. Mater. Res.*, 2022, **52** , 103 —128.

4 S. S. Park, Y. Tulchinsky, and M. Dincă, *J. Am. Chem. Soc.*, 2017, **139**, 13260–13263.

5 J. Evans, C. A. Vincent and P. G. Bruce, *Polymer*, 1987, **28**, 2324-2328.

6 H. Yang and N. Wu, *Energy Sci. Eng.*, 2022, **10**, 1643–1671.

7 X. Duan, Y. Ouyang, Q. Zeng, S. Ma, Z. Kong, A. Chen, Z. He, T. Yang and Q. Zhang, I*norg. Chem*., 2021, **60**, 11032–11037.

8 Y. Xu, R. Zhao, J. Fang, Z. Liang, L. Gao, J. Bian, J. Zhu and Y. Zhao, *Front. Chem.*, 2022, **10**, 1013965.

9 Q. Zeng, L. Shi, J. Wang, X. Zha, W. Yang and Y. Yang, *J. Energy Storage*, 2025, **110**, 115314.

10 Y. Sun , Z. Hu , D. Zhao and K. Zeng , *ACS Appl. Mater. Interfaces*, 2017, **9** , 32202 —32210.

11 Z. Wang , K. M. Schmalbach , R. L. Combs , Y. Chen , R. L. Penn , N. A. Mara and A. Stein , *ACS Appl. Mater. Interfaces*, 2020, **12** , 49971 —49981

12 P. Ying , J. Zhang and Z. Zhong , *J. Phys. Chem. C*, 2021, **125** , 12991 —13001

13 K. Banlusan and A. Strachan , *J. Chem. Phys.*, 2017, **146** , 184705

14 E. Alvarez, N. Guillou, C. Martineau, B. Bueken, B. Van de Voorde, C. Le Guillouzer, P. Fabry, F. Nouar, F. Taulelle, D. de Vos, J. Chang, K. H. Cho, N. Ramsahye, T. Devic, M. Daturi, G. Maurin and C. Serre, *Angew Chem Int Ed*, 2015, **54**, 3664–3668.
